# IL-6 signaling accelerates iron overload by upregulating DMT1 in endothelial cells to promote aortic dissection

**DOI:** 10.7150/ijbs.99511

**Published:** 2024-08-06

**Authors:** Qiang Xie, Jianji Wang, Runqiao Li, Hao Liu, Yongliang Zhong, Qinfeng Xu, Yipeng Ge, Chengnan Li, Lizhong Sun, Junming Zhu

**Affiliations:** 1 Department of Cardiovascular Surgery, Beijing Aortic Disease Center, Beijing Anzhen Hospital, Capital Medical University, Beijing 100029, China.; 2 Department of Thoracic Surgery, Ruijin Hospital, Shanghai Jiao Tong University School of Medicine, Shanghai 200025, China.

**Keywords:** aortic dissection, iron overload, divalent metal transporter 1, endoplasmic reticulum stress, single-cell RNA sequencing

## Abstract

Aortic dissection (AD), caused by tearing of the intima and avulsion of the aortic media, is a severe threat to patient life and organ function. Iron is closely related to dissection formation and organ injury, but the mechanism of iron ion transport disorder in endothelial cells (ECs) remains unclear. We identified the characteristic EC of dissection with iron overload by single-cell RNA sequencing data. After intersecting iron homeostasis and differentially expressed genes, it was found that hypoxia-inducible factor-1α (HIF-1α) and divalent metal transporter 1 (DMT1) are key genes for iron ion disorder. Subsequently, IL-6R was identified as an essential reason for the JAK-STAT activation, a classical iron regulation pathway, through further intersection and validation. In *in vivo* and *in vitro*, both high IL-6 receptor expression and elevated IL-6 levels promote JAK1-STAT3 phosphorylation, leading to increased HIF-1α protein levels. Elevated HIF-1α binds explicitly to the 5'-UTR sequence of the DMT1 gene and transcriptionally promotes DMT1 expression, thereby increasing Fe^2+^ accumulation and endoplasmic reticulum stress (ERS). Blocking IL-6R and free iron with deferoxamine and tocilizumab significantly prolonged survival and reduced aortic and organ damage in dissection mice. A comparison of perioperative data between AD patients and others revealed that high free iron, IL-6, and ERS levels are characteristics of AD patients and are correlated with prognosis. In conclusion, activated IL-6/JAK1/STAT3 signaling axis up-regulates DMT1 expression by increasing HIF-1α, thereby increasing intracellular Fe^2+^ accumulation and tissue injury, which suggests a potential therapeutic target for AD.

## Introduction

Aortic dissection (AD) is an injury to the aortic wall, including intimal rupture and middle aortic avulsion^1^. Surgery is the only treatment for acute aortic dissection (AAD), which can be life-threatening after onset^2^. The course of AD involves pathological processes with severe organ dysfunction, especially acute kidney injury (AKI), which is caused by inflammatory factors and harmful substances with redox activity^3^-^5^.

Systemic iron ions are safely stored as bound iron, recognized by the transferrin receptor (TfR) endocytosed, reduced to Fe^2+^, and transported to the cytoplasm via divalent metal transporter 1 (DMT1) or Zrt/Irt-related proteins 14/8 (ZIP-14/8)^3^. Then, it binds to the ferritin heavy chain (FTH1) and light chain (FTL) for safe storage (iron load marker)^6^. Circulating free iron that does not bind to transferrin, hemoglobin, or ferritin is called non-transferrin-bound iron, which has catalytic cytotoxicity^7^. Abnormal free iron ion content and transporter expression, such as DMT1, can lead to ferrous ion metabolism disorders and diseases^8^.

Early persistent endoplasmic reticulum stress (ERS) in smooth muscle and endothelial cells (ECs) is one of the leading causes of AD. ERS disrupts the association of endoplasmic reticulum (ER) with mitochondria, increasing mitochondrial Ca^2+^ (mito-Ca^2+^) and superoxide (mitoSOX)^9^,^10^. Especially during the acute phase of AD, ERS peaks induced by excessive inflammation and internal environmental disorders, exacerbating progression and organ damage^4^,^11^.

The JAK/STAT pathway is the primary regulation for iron balance, which can be activated by interleukin, interferon (IFN), and colony-stimulating factor (CSF)^12^. The canonical JAK/STAT iron regulation exacerbates intracellular iron ion accumulation by promoting hepcidin release, degrading the only iron exporter, ferroportin (FPN)^13^. Iron overload can produce the Fenton reaction, which produces abundant oxygen radicals and induces ERS^14^. Although the JAK/STAT pathway has been rarely reported to promote iron ions into cells, exploring such a noncanonical mechanism is of great significance.

This research pioneered the discovery of a specific iron-overload subset of human aortic endothelial cells (HAECs) in dissecting aorta by single-cell RNA sequencing (scRNA-seq) data. The noncanonical mechanism by which high DMT1 expression and iron ion dysregulation in HAECs induced by activated IL-6R/JAK1/STAT3 signaling axis were elucidated in this study. Our results provided new therapeutic insights for AD, which can currently be treated only surgically.

## Materials and methods

### ScRNA-seq data dimension reduction and pseudotime analysis

The scRNA-seq data (www.ncbi.nlm.nih.gov/bioproject/PRJNA882355) obtained from Bioproject, which included aortic tissue of six AD patients and three heart transplant donors, were analyzed via the “Seurat” package. The percentages of mitochondria and rRNA were calculated using the PercentageFeatureSet function. After filtration, the first 30 principal component analyses (PCAs) were used to construct a uniform manifold approximation and projection (UMAP) for visualization. Single-cell pseudotime and trajectory analyses were performed with the “Monocle3” package^15^,^16^.

### Bulk mRNA-seq data collection and analysis

Quantification, qualification, library preparation, and RNA sequencing of tissue RNA were completed by Novogene Co. (Beijing, China). Aortic tissue from three AD patients and three heart transplant donors, as well as from the aortas of six control and six AD mice at the acute stage, were used for library preparation.

### Identification of differential expression genes (DEGs) and enrichment analysis

The genes with a |log2 value (FC)| > 0.5 and a false discovery rate (FDR) < 0.05 after FDR adjustment were considered DEGs. Gene Ontology (GO) and Kyoto Encyclopedia of Genes and Genomes (KEGG) enrichment analyses of DEGs were performed using the online Database for Annotation, Visualization, and Integrated Discovery tool^17^,^18^. An adjusted* P* < 0.05, obtained using the Benjamini & Hochberg method, was considered to indicate a statistically significant difference^19^.

### Cells and reagents

The aortic tissues used were discarded during heart transplantation. The endothelial tissue was dissected along the inner surface of the aortic wall and washed in normal saline. Tissues were cut into evenly sized (1-2 mm) and digested with 0.2% type I collagenase (Sigma, Missouri, USA; Cat. C0130) at 37℃ for 50 minutes. Then, cells were filtered and centrifuged (1000 r/min, 5 min). HAECs were re-suspended in ECM medium (ScienCell, California, USA; Cat. 1001) and continued to be cultured. In this study, HAECs were used between the second and fourth generations^20^.

The pCMV-IL6R was purchased from SHHE-Bio Co. (Shanghai, China; Cat. P46637). AZD-1480 (Cat. HY-10193) was used for inhibiting JAK1 activation, SYP-5 (Cat. HY-100693) for inhibiting hypoxia-inducible factor-1α (HIF-1α), FG-4592 (Cat. HY-13426) for increasing HIF-1α, DMT1 blocker 1 (C-6F; Cat. HY-126301) for blocking DMT1, deferoxamine (DFO; Cat. HY-B1625) for chelating iron ions, tocilizumab (TCZ; Cat. HY-P9917) for blocking interleukin-6 receptor (IL-6R), ferric ammonium citrate (FAC; Cat. HY-B1645) and interleukin-6 (IL-6) recombinant protein (Cat. HY-P7044) for simulating the acute phase. These reagents were purchased from MCE Co. (New Jersey, USA).

### Real-time polymerase chain reaction (RT‒PCR)

The total RNA of tissue or cell was extracted by the TRIzol reagent (Invitrogen, CA, USA, Cat. 15596026). Reverse transcription was performed using the kit (Promega, USA, Cat. A5001). RT‒PCR was performed using SYBR Master Mix (Vazyme, China, Cat. Q712). The relative mRNA expression was calculated using the ^-△△^Ct method relative to those of GAPDH. The primers are listed in **Table S1**.

### Flow cytometry assay

Stimulated cells were collected, and an appropriate number of cells were stained according to the instructions of the AV-PI apoptosis kit (BD, California, USA; Cat. 556547) or reactive oxygen species (ROS) assay kit (Beyotime, Shanghai, China; Cat. S0033). The stained cells were collected with the flow cytometer (BD, California, USA; Cat. LSR Fortessa).

### Confocal fluorescence imaging

FerroOrange (Dojindo, Japan; Cat. F374) was used to label Fe^2+^. Mito-Ca^2+^ and mitoSOX were labeled with indicators (Yeasen, Shanghai, China; Cat. 40778 and 40776). Mito-Tracker and ER-Tracker (Beyotime, Shanghai, China; Cat. C1048 and C1042) were used for organelle staining. Cell nuclei were stained with Hoechst 33258 (Beyotime, Shanghai, China; Cat. C1011). Fluorescence images were taken using a laser scanning confocal microscope (Leica, Wetzlar, Germany; Cat. TCS SP5).

### Paraffin-embedded sections and section staining

The aorta or kidney tissue was fixed in 4% paraformaldehyde, embedded in paraffin, and then cut into 4 μm thick slices. The sections were stained with an immunohistochemical (IHC) staining kit (ZSGB-Bio, Beijing, China; Cat. SAP-9100). Multicolor immunofluorescence (multi-IF) staining was performed using a multiplex IHC kit (AlphaTSA, Beijing, China; Cat. ATX37100031). The antibodies used are listed in **Table S2**. Verhoeff-van Gieson (EVG) staining (Cat. G1597), prussian blue (PB) iron staining (Cat. G1422), and Periodic acid-Schiff (PAS) staining (Cat. G1281) reagents were purchased from Solarbio Co. (Beijing, China).

### Western blot assay (WB)

Equal amounts of quantified protein were separated via SDS‒PAGE and transferred onto a PVDF membrane. The membrane was blocked in 5% BSA for 1 hour at room temperature and incubated overnight at 4°C in the primary antibody with suitable concentration (**Table S2**). Infrared-labeled secondary antibodies were used for detection on an Odyssey Infrared Imaging System.

### Transcription repression assay

Actinomycin D (Act-D; MCE, New Jersey, USA; Cat. HY-17559) was added to cells (2mg/ml) after overexpressing HIF-1α. The cells were collected at 0, 0.5, 1, and 2 hours, then the DMT1 mRNA level was assessed.

### Chromatin immunoprecipitation (ChIP) assay

After the cell DNA was fragmented via ultrasound, the remaining steps were performed according to a ChIP assay instruction (CST, Massachusetts, USA, Cat. #9003). DNA fragments were immunoprecipitated with HIF-1α antibody, and the different sequences were amplified with specific primers (**Table S1**).

### Dual-luciferase assay

Full-length and mutated DMT1 promoter sequences were cloned and inserted into the pGL3-basic vector, which was subsequently transfected into cells using Lipofectamine 3000 (Thermo Fisher, USA, Cat. L3000015). The pRL-TK Renilla plasmid was used for normalization (Promega, USA, Cat. E1910). The primers for plasmid construction are listed in **Table S1**.

### Pulldown assay

Special biotin-labeled primers (**Table S1**) were used to amplify DMT1 promoter probes containing different sites. The probes and beads were incubated with nuclear protein (Thermo Fisher, Massachusetts, USA, Cat. 20164). Then, equal amounts of purified protein from different groups were used for WB assay.

### Clinical data and specimens

The aortic tissues used were discarded during heart transplantation and AD surgery. Informed consent for specimen use was obtained from the patients and approved by the Medical Ethical Committee of Capital Medical University (KS2019016). From September 2021 to March 2022, AD patients undergoing open operations and other patients requiring aortic surgery at our center were included. Patients with malignancy, pregnancy, end-stage renal disease, cardiovascular surgery history, and those aged under 18 or over 70 years were excluded. Postoperative serious adverse events (SAEs) were defined as exploratory thoracotomy, stroke, paraplegia, renal replacement therapy, AKI-Stage 3, prolonged hospital stay, and mechanical ventilation. The use of observational data was approved by the Medical Ethical Committee of Beijing Anzhen Hospital of Capital Medical University (2023191X).

### Animal model

Wild-type C57BL/6 mice were purchased from HFK-Bio Sci Co. (Beijing, China) and randomly grouped. All experiments were reviewed and approved by the Medical Sciences Animal Protection and Use Committee of Capital Medical University (AEEI-2022-033). Four-week-old male mice were fed a routine diet or feed containing 0.15% β-aminopropionitrile (BAPN; Sigma, Missouri, USA) for four weeks. Then, the mice were subcutaneously implanted with mini-osmotic pumps (Alzet, California, USA), administered 1 μg/kg/min Angiotensin II (Ang II; Sigma, Missouri, USA), and euthanized 48 hours after implantation. Aortal diameter was measured via ultrasound (VisualSonics, Canada; Cat. Vevo2100). Blood and tissue samples were collected for further analysis.

### Biochemical criteria for serum and tissue analyses

Blood samples were centrifuged at 3000 rpm at 4°C for 5 minutes, then the serum was collected and stored at -80°C. Enzyme-linked immunosorbent assay (ELISA) kits used are listed in **Table S3**. The tissue contents of Fe^2+^ and malondialdehyde (MDA) were measured (Solarbio, Beijing, China; Cat. BC5415 and BC0020). Data were obtained by a multifunctional microplate reader (Dynex, Minnesota, USA; Cat. Spectra MR).

### Statistical analysis

Data are shown as the mean ± standard deviation (SD) and analyzed with GraphPad Prism version 9. Comparisons among groups were performed using one-way ANOVA analysis. The Kaplan-Meier method estimated the overall survival of mice. For clinical data, continuous variables are analyzed using the Student's t-test or Mann-Whitney U test; the categorical variables are presented as frequencies and percentages, compared by χ2 analysis or Fisher's exact test. Pearson correlation analysis was used for linear correlation analysis. Logistic regression was used to identify the risk factors for SAEs. Preoperative and operation-related variables with *p*-values < 0.20 in univariable regression were included in multivariable regression. All the experiments were repeated at least three times, and a two-tailed *p*-value < 0.05 was considered statistically significant.

## Results

### scRNA-seq analysis revealed iron overload in HAECs

**Figure 1:**
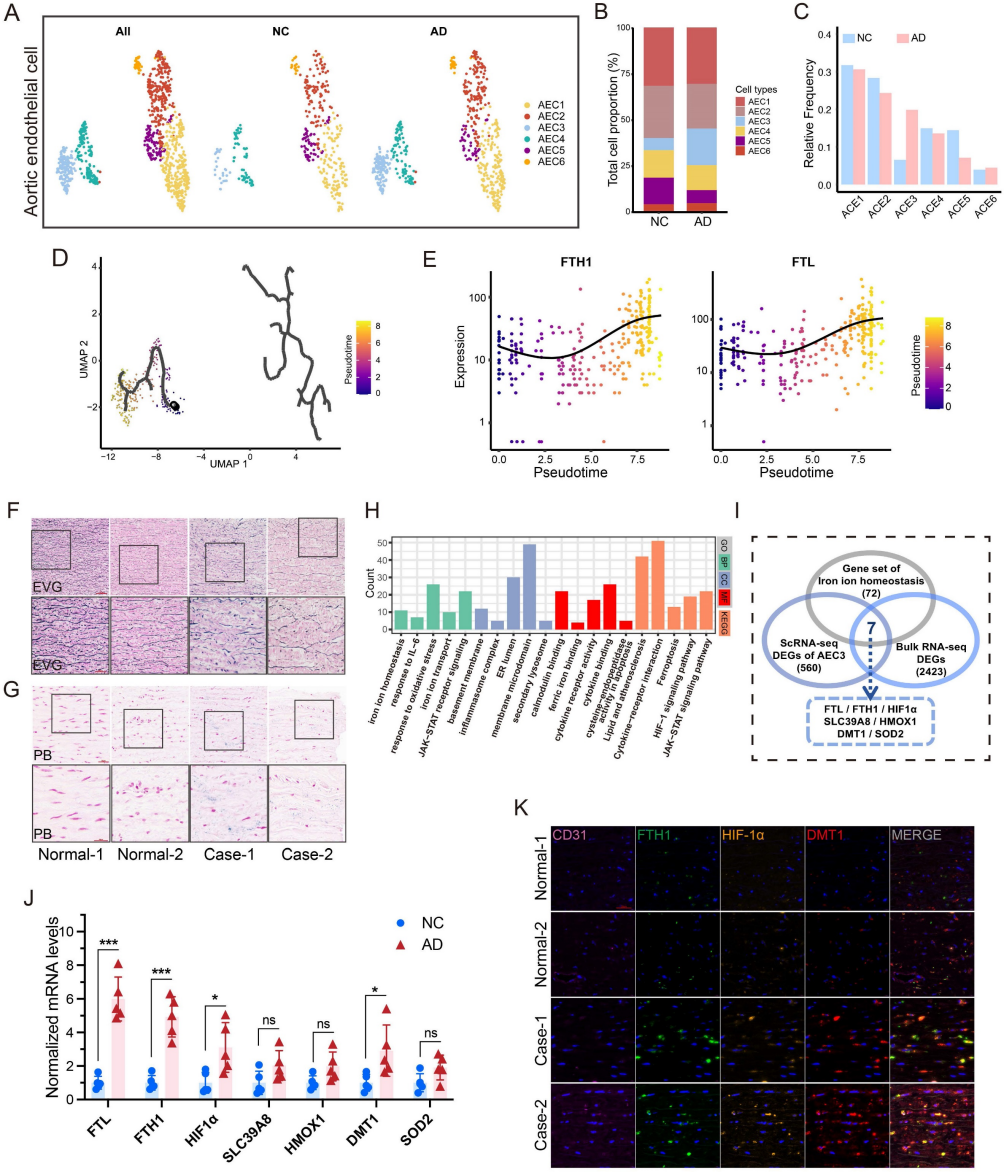
Endothelial cells from aortic dissection showed evident iron overload. (**A**) UMAP plot of AEC types. (**B** and **C**) Proportions of different AECs. (**D**) Pseudotime and trajectory analysis of AEC development. (**E**) Expression profiles of representative markers (*FTH1* and *FTL*) at pseudotime. (**F**) EVG staining of patient and transplant donor aortas. (**G**) PB iron staining of aortic sections. (**H**) GO and KEGG analyses of bulk RNA-seq DEGs of AD patient and transplant donor aortas. (**I**) Venn diagram of the comparison between iron ion homeostasis genes and two sets of EDGs. (**J**) RT-PCR assays showed the key gene expressions in dissection and donor aortic tissues. (**K**) Multi-IF exhibited the protein levels of key genes (CD31, endothelial cell marker). (**L**) GSEA analysis indicated the significant enrichment of the JAK-STAT pathway. Data are presented as representative images or means ± SDs of three independent assays. *P < 0.05, **P < 0.01, ***P < 0.001, ns, not significant.

After quality control and batch correction, 83893 cells were obtained for downstream analysis (**Fig. S1A**-**B**). Seven cell types were identified (**Fig. S1C**-**D**). After dimensionality reduction for ECs, four types were identified (aortic, capillary, vein, and lymphatic ECs) (**Fig. S1E**-**I**)^21^. After further dimensionality reduction in aortic ECs (AECs) (**Fig. 1A**), an increase in AEC3 proportion was found in AD group (**Fig. 1B**-**C**). The pseudotime trajectories of AECs are presented in **Fig. 1D**. *FTL* and *FTH1*, essential markers of iron content, were representative of AECs differentiation (**Fig. 1E**). *FTL* was one of the most upregulated genes in AEC3 (**Fig. S1J**). The enrichment analyses of AEC3 DEGs showed that iron homeostasis, metal ion transport, and ROS metabolism were among the significantly enriched items (**Fig. S2A**).

### HIF-1α and DMT1 are key genes of iron overload

Dissecting aortic tissue exhibited obvious elastic fiber fracture and more iron deposition, confirming the iron overload feature (**Fig. 1F**-**G**). Aortic tissues from AD patients and transplant donors were used for bulk mRNA-seq analysis to confirm database results (**Fig. S2B**). GO and KEGG analyses revealed that iron ion transport, homeostasis, and JAK-STAT pathways are one of the most important pathways (**Fig. 1H**). Since iron overload is a characteristic of dissection, the iron ion homeostasis-related genes, EDGs of AEC3, and EDGs of bulk mRNA-seq were intersected to obtain key genes. The results showed that *FTL*, *FTH1*, *HIF-1α*, *SLC39A8*, *HMOX1*, *SLC11A2* (*DMT1*), and *SOD2* mRNA were all highly expressed (**Fig. 1I**). RT-PCR confirmed that *FTL*, *FTH1*, *HIF-1α,* and *DMT1* mRNA were actually highly expressed in aortas with dissection (**Fig. 1J**). Multi-IF confirmed the high FTH1, HIF-1α, and DMT1 protein levels in dissecting aortas (**Fig. 1K**).

### Features are more pronounced in acute dissection

**Figure 2:**
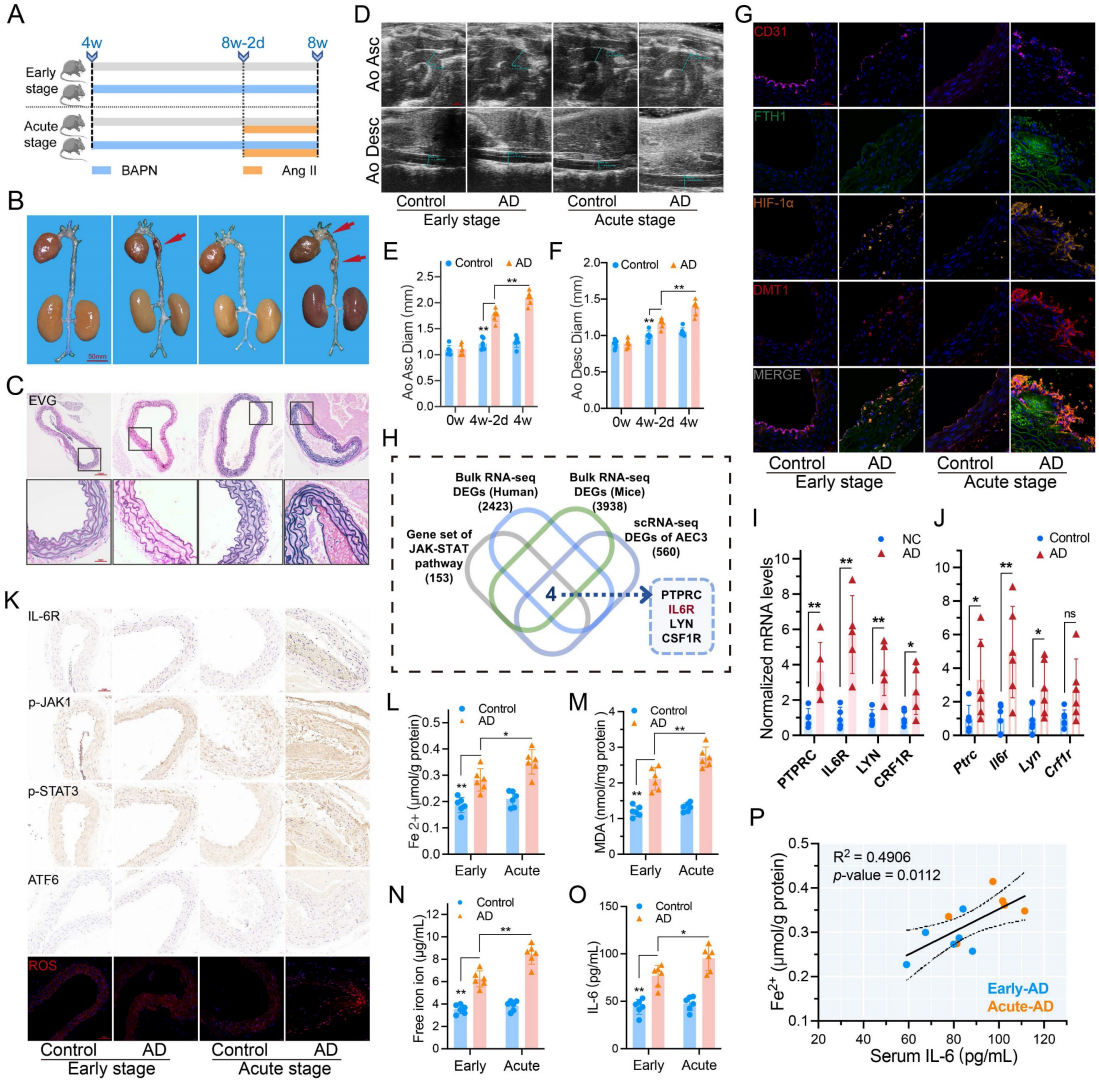
IL-6R and JAK-STAT pathway promoted dissection progression. (**A**) Acute stage was induced by Ang II (Six mice per group). (**B**) Gross anatomical views of mouse aortas. (**C**) EVG staining of dissecting aortas. (**D**) Ultrasound images of the ascending and descending aortas. (**E**, **F**) Comparison of ascending and descending aorta diameters. (**G**) Multi-IF exhibited the protein levels of essential genes. (**H**) Venn diagram of the comparison between JAK-STAT pathway genes and three sets of EDGs. (**I**, **J**) RT-PCR assays showed the intersecting gene expressions in patients and mice aortas with dissection. (**K**) IHC staining (IL-6R, p-JAK1, p-STAT3, and ATF6) and ROS staining in mouse aortic sections. (**L**, **M**) Contents of Fe^2+^ and MDA in mouse aortas. (**N**, **O**) Contents of free iron ion and IL-6 in mouse serums. (**P**) Correlation between serum IL-6 level and aortic Fe^2+^ content in mice with dissection. Data are presented as representative images or means ± SDs of three independent assays. *P < 0.05, **P < 0.01, ***P < 0.001, ns, not significant.

Ang II was used to induce the acute phase of AD (**Fig. 2A**). Short-term Ang II administration markedly exacerbated early BAPN-induced AD (**Fig. 2B**-**C**). Mice with AAD had further dilated ascending and descending aortas (**Fig. 2D**-**F**). Multi-IF confirmed high FTH, HIF-1α, and DMT1 expression in dissecting aorta, which was even higher in AAD (**Fig. 2G**).

### IL-6R is the key to JAK-STAT upregulation

Aortas from AAD and control mice were used for bulk mRNA-seq analysis (**Fig. S2C**). The JAK-STAT pathway-related genes, EDGs of scRNA-seq, and EDGs of human and mice bulk mRNA-seq were intersected to obtain essential genes for JAK-STAT pathway regulation. The results showed that *PTPRC*, *IL6R*, *LYN*, and *CSF1R* mRNA were all highly expressed (**Fig. 2H**). The confirmatory RT-PCR assays showed that IL-6R mRNA expression had the highest difference among the four candidates (**Fig. 2I**-**J**). IHC staining revealed elevated IL-6R, p-JAK1, and p-STAT3 levels in dissecting aortas. Levels of ERS markers, activating transcription factor 6 (ATF6) and ROS of ECs, were also characterized as higher in the acute phase (**Fig. 2K**).

### IL-6 is correlated with iron ion content and metabolism

The Fe^2+^ and MDA contents increased in dissecting aortas, especially in acute, as did the serum free iron ion and IL-6 levels (**Fig. 2L**-**O**). Serum levels of hepcidin, creatinine, Kim-1, and ROS exhibited similar characteristics (**Fig. S3A**-**D**). Free iron and hepcidin levels confirmed circulating iron overload. Moreover, excessive IL-6 during the acute phase further activates the IL-6R/JAK/STAT pathway, leading to worse progression and organ damage.

The aortic Fe^2+^ content was positively correlated with serum IL-6 in AD mice (R^2^ = 0.4906, P = 0.0112) (**Fig. 2P**). GO analysis of AD mice revealed that metal ion homeostasis, bivalent cation homeostasis, and metal ion transporters' biological functions and pathways were significantly enriched in the high IL-6 group (**Fig. S2D**).

Taken together, the potential links of the key genes HIF-1α and DMT1 for iron overload, as well as the IL-6R/JAK/STAT pathway that affects aortic iron content and metabolism, are the focus of this research.

### High IL-6R increased Fe^2+^ and ERS by DMT1

**Figure 3:**
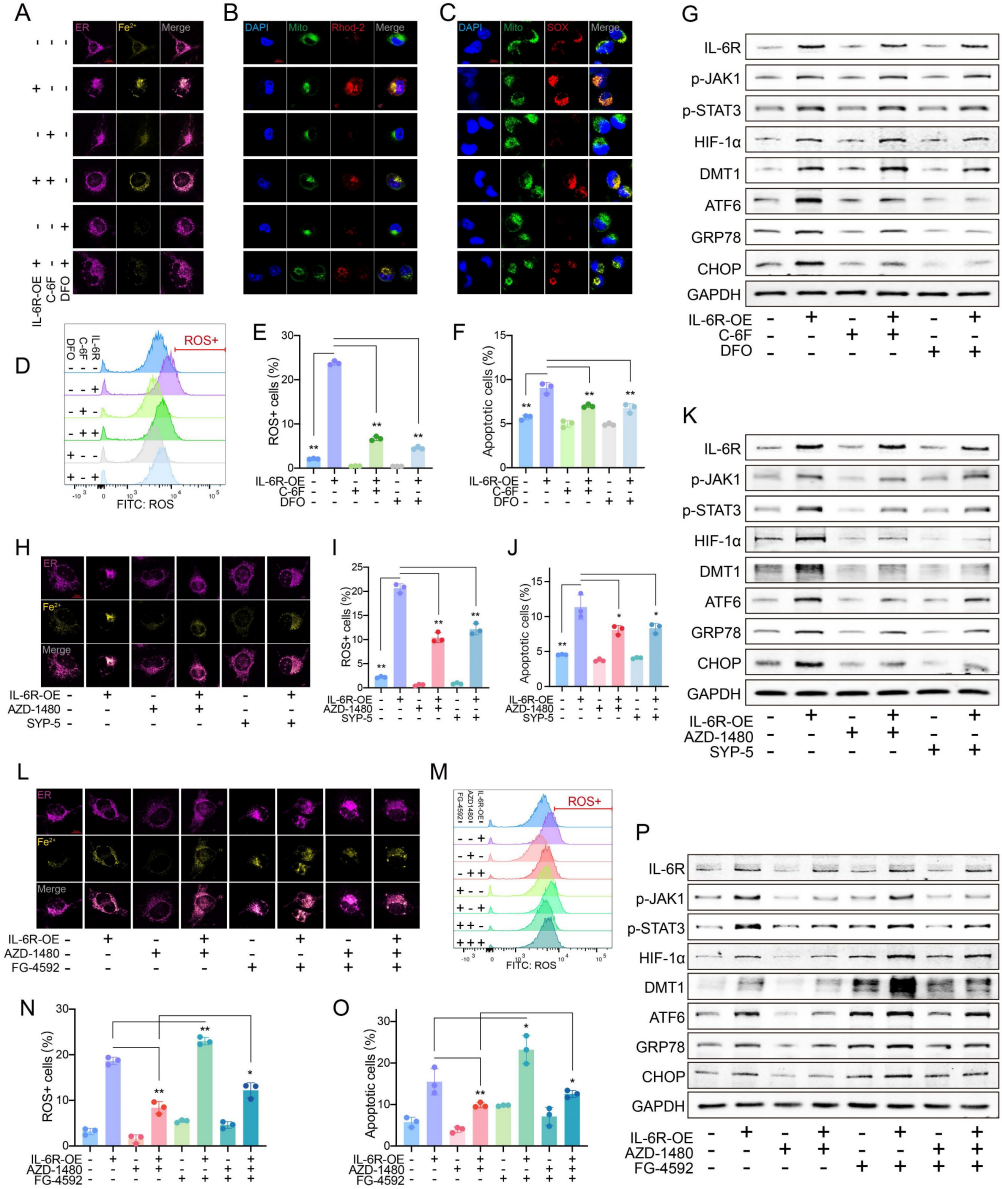
High IL-6R upregulated DMT1 to induce iron overload and ERS. (**A**) C-6F (5 μM, 24 h) and DFO (100 μM, 24 h) decreased the Fe^2+^ content in HAECs. (**B**) Fluorescence staining of mito-Ca^2+^. (**C**) Fluorescence staining of mitoSOX. (**D**, **E**) Flow cytometry detected ROS levels in HAECs. (**F**) Flow cytometry detected apoptosis levels. (**G**) WB showed IL-6R, p-JAK1, p-STAT3, HIF-1α, DMT1, and ERS pathway levels. (**H**) AZD-1480 (1 μM, 24 h) and SYP-5 (50 μM, 24 h) decreased the intracellular Fe^2+^ content. (**I**, **J**) Flow cytometry detected ROS and apoptosis levels. (**K**) WB showed IL-6R, p-JAK1, p-STAT3, HIF-1α, DMT1, and ERS pathway levels. (**L**) AZD-1480 (1 μM, 24 h) and FG-4592 (50 μM, 24 h) changed the Fe^2+^ content. Flow cytometry detected ROS (**M**, **N**), and apoptosis (**O**) levels. (**P**) WB showed IL-6R, p-JAK1, p-STAT3, HIF-1α, DMT1, and ERS pathway levels. Data are presented as representative images or means ± SDs of three independent assays. *P < 0.05, **P < 0.01, ***P < 0.001, ns, not significant.

High IL-6R expression markedly increased the Fe^2+^ level in HAECs, which was inhibited by C-6F, especially DFO (**Fig. 3A**). An increase in IL-6R resulted in high mito-Ca^2+^ and mitoSOX (**Fig. 3B**-**C**). ROS levels increased significantly as IL-6R overexpression (**Fig. 3D**-**E**). More IL-6R came with increased HAEC apoptosis (**Fig. 3F**, **Fig. S4A**). The inhibition from DFO was greater than that from C-6F. IL-6R overexpression upregulated p-JAK1, p-STAT3, HIF-1α, DMT1, ATF6, glucose regulated protein (GRP78) and CHOP levels. C-6F and DFO alleviated ERS but did not alter JAK-STAT phosphorylation or HIF-1α/DMT1 levels (**Fig. 3G**).

### HIF-1α/DMT1 as the downstream of JAK-STAT

Elevated Fe^2+^, ROS, and apoptosis due to IL-6R overexpression were inhibited by inhibitors of JAK1 phosphorylation (AZD-1480) and HIF-1α (SYP-5) (**Fig. 3H-K**, **Fig. S4B-E**). Inhibition of JAK1 phosphorylation resulted in decreased downstream marker levels. After HIF-1α suppression, IL-6R-induced JAK-STAT phosphorylation did not change significantly, but DMT1 and ERS marker levels decreased (**Fig. 3K**).

After controlling JAK1 activation, FG-4592 was used to increase the HIF-1α accumulation. HIF-1α can significantly increase the decreased levels of Fe^2+^, mito-Ca^2+^, mitoSOX, ROS, and apoptosis due to JAK dephosphorylation (**Fig. 3L**-**O**, **Fig. S4F**-**H**). High HIF-1α had no noticeable effect on JAK-STAT but partially reversed the decrease in DMT1 and ERS marker levels due to JAK dephosphorylation (**Fig. 3P**).

### IL-6 increased sensitivity of HAECs to FAC by upregulating DMT1

**Figure 4:**
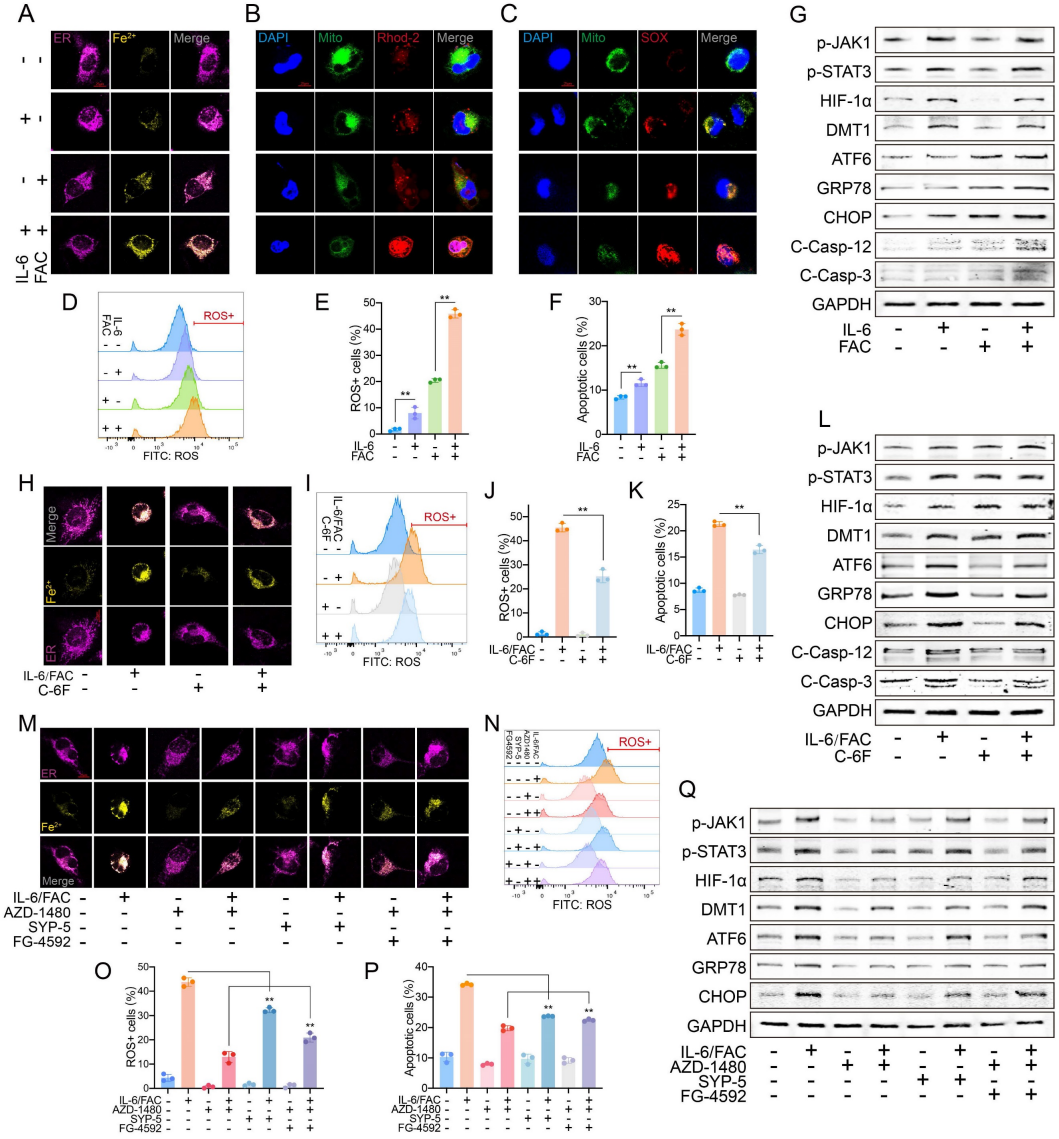
IL-6 sensitized FAC-induced iron overload by upregulating DMT1 level. (**A**) IL-6 (50 ng/ml, 24 h) and FAC (12 mg/L, 24 h) increased the Fe^2+^ content in HAECs. (**B**) Fluorescence staining of mito-Ca^2+^. (**C**) Fluorescence staining of mitoSOX. (**D**, **E**) Flow cytometry detected ROS levels. (**F**) Flow cytometry detected apoptosis levels. (**G**) WB showed p-JAK1, p-STAT3, HIF-1α, DMT1, and ERS pathway levels. (**H**) C-6F (5 μM, 24 h) decreased the intracellular Fe^2+^ content. Flow cytometry detected ROS (**I**, **J**) and apoptosis (**K**) levels. (**L**) WB showed p-JAK1, p-STAT3, HIF-1α, DMT1, and ERS pathway levels. (**M**) AZD-1480 (1 μM, 24 h), SYP-5 (50 μM, 24 h), and FG-4592 (50 μM, 24 h) changed the Fe^2+^ content. Flow cytometry detected ROS (**N, O**), and apoptosis (**P**) levels. (**Q**) WB showed IL-6R, p-JAK1, p-STAT3, HIF-1α, DMT1, and ERS pathway levels. Data are presented as representative images or means ± SDs. *P < 0.05, **P < 0.01, ***P < 0.001, ns, not significant.

*In vitro*, IL-6 and FAC were used to simulate circulating excessive IL-6 and free iron in AAD. A high extracellular free iron ion increased the intracellular Fe^2+^, especially with IL-6 combination (**Fig. 4A**). Similarly, IL-6 further increased the FAC-induced high mito-Ca^2+^ and mitoSOX (**Fig. 4B**-**C**). IL-6 alone did not induce ROS or apoptosis as much as FAC did, but IL-6 clearly sensitized HAECs to FAC-induced damage (**Fig. 4D**-**F**, **Fig. S5A**). WB assays showed that excessive IL-6 activated JAK-STAT signaling and increased HIF-1α and DMT1 expression. However, FAC did not phosphorylate JAK-STAT, but ERS levels were significantly increased. The combination resulted in the highest ERS level (**Fig. 4G**).

Blocking DMT1 decreased the increase in Fe^2+^, mito-Ca^2+^, and mitoSOX induced by IL-6 and FAC (**Fig. 4 H**, **Fig. S5B**-**C**); it also inhibited ROS and apoptosis (**Fig. 4I-K**, **Fig. S5D**). WB showed that blocking DMT1 did not greatly alter upstream expression, but ERS marker levels decreased sharply (**Fig. 4L**).

After inhibiting p-JAK or HIF-1α, the Fe^2+^, mito-Ca^2+^, and mitoSOX levels markedly decreased. After JAK1 dephosphorylation and HIF-1α upregulation, the metal ion and mitoSOX levels differed notably (**Fig. 4M**, **Fig. S5E**-**F**). Similar results were observed in ROS and apoptosis assays (**Fig. 4N-P**, **Fig. S5G**). WB showed that downstream signaling was downregulated after JAK dephosphorylation. Inhibiting HIF-1α decreased DMT1 and ERS but did not affect JAK-STAT activation. After inhibiting p-JAK and increasing HIF-1α, the DMT1 and ERS marker levels increased, but JAK-STAT was still suppressed (**Fig. 4Q**). Overall, JAK-STAT is upstream of HIF-1α/DMT1, and HIF-1α directly affects DMT1 expression.

### HIF-1α increased DMT1 expression through transcriptional regulation

**Figure 5:**
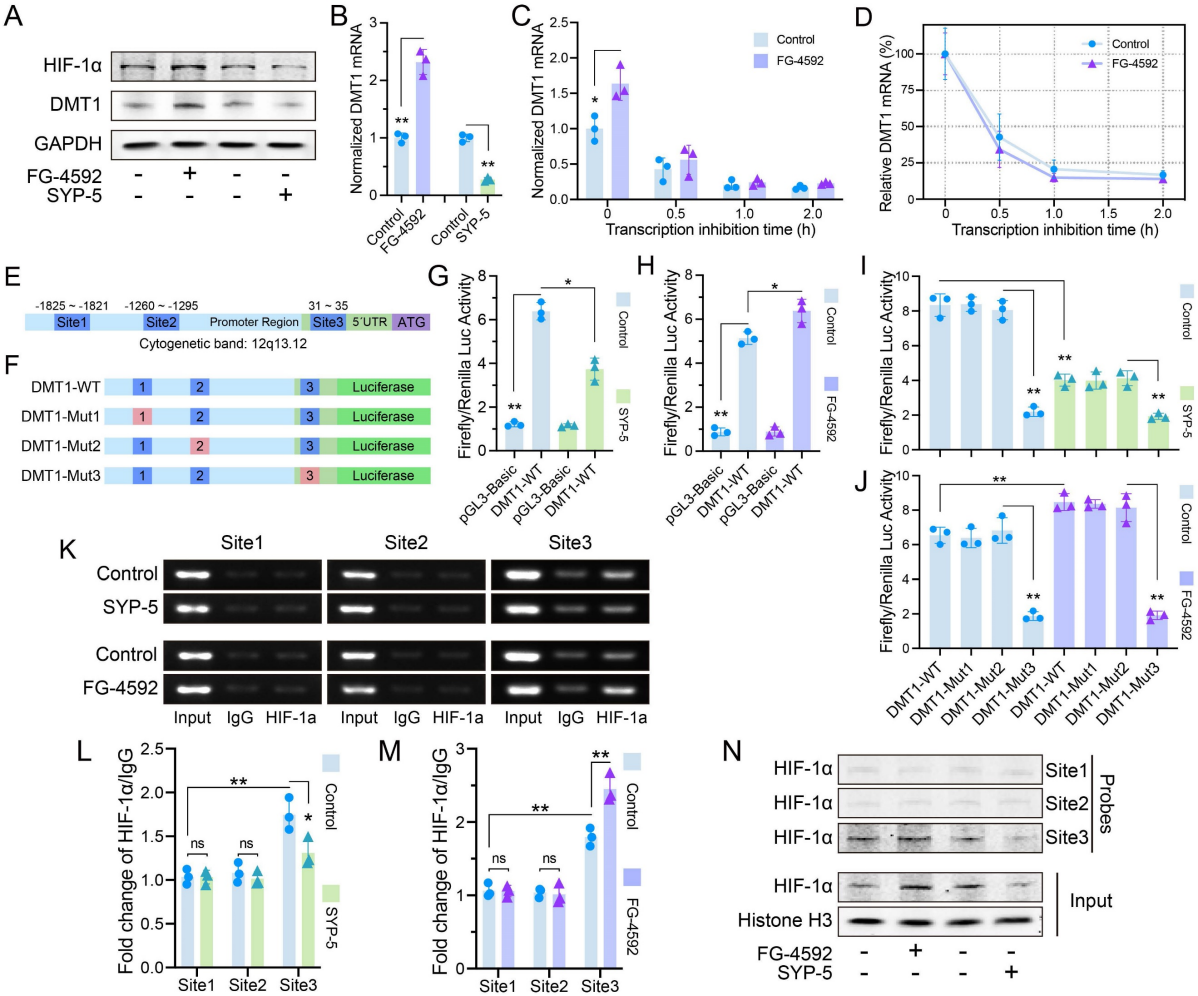
HIF-1α increased DMT1 expression through transcriptional regulation. (**A**) Protein level of DMT1 changed with HIF-1α level (FG-4592: 50 μM, 24 h; SYP-5: 50 μM, 24 h). (**B**) Level of DMT1 mRNA changed with HIF-1α expression. (**C**, **D**) Half-life of DMT1 mRNA was compared at different HIF-1α protein levels (Treatment with 50 μM of FG-4592 for 24 h, then treat with 2mg/ml Act-D for 0.5, 1, and 2 h). (**E**) Three potential HIF-1α binding sites in DMT1 promoter region. (**F**) DMT1-WT and mutant plasmids for luciferase assay. (**G**, **H**) Luciferase activity of DMT1-WT and pGL3-Basic plasmids at different HIF-1α levels. (**I**, **J**) Luciferase activity of DMT1-WT and mutant plasmids. (**K**) ChIP assays verified that HIF-1α has more binding to Site 3. (**L**, **M**) Quantification of HIF-1α relative to IgG in ChIP. (**N**) Pulldown assays verified that Site 3 probe bound to HIF-1α. Data are presented as representative images or means ± SDs. *P < 0.05, **P < 0.01, ***P < 0.001, ns, not significant.

HIF-1α accumulation in JAK1-STAT3 signaling has been widely reported, but we found that DMT1 expression changed with HIF-1α^22^. DMT1 protein and mRNA levels were consistent with HIF-1α protein (**Fig. 5A**-**B**). The half-life of DMT1 mRNA did not change evidently after HIF-1α accumulation, suggesting that regulation may occur by increasing transcription rather than stabilizing DMT1 mRNA (**Fig. 5C**-**D**). Three potential HIF-1α binding sequences (-A/GCGTG-) were found in DMT1 promoter region (**Fig. 5E**). A wild-type plasmid containing complete sequences (DMT1-WT) and three mutant plasmids were constructed for luciferase assays (**Fig. 5F**). Luciferase activity of DMT1-WT was greater than that of vector, and changed with the HIF-1α level (**Fig. 5G**-**H**). Luciferase activity decreased after mutation at Site 3 (**Fig. 5I**-**J**). ChIP assays confirmed the increased binding of HIF-1α to Site3 fragment (**Fig. 5K**-**M**). Pulldown assays revealed that the Site 3 probe bound to more HIF-1α protein (**Fig. 5N**).

### Inhibition of IL-6R and free iron alleviated dissection progression

**Figure 6:**
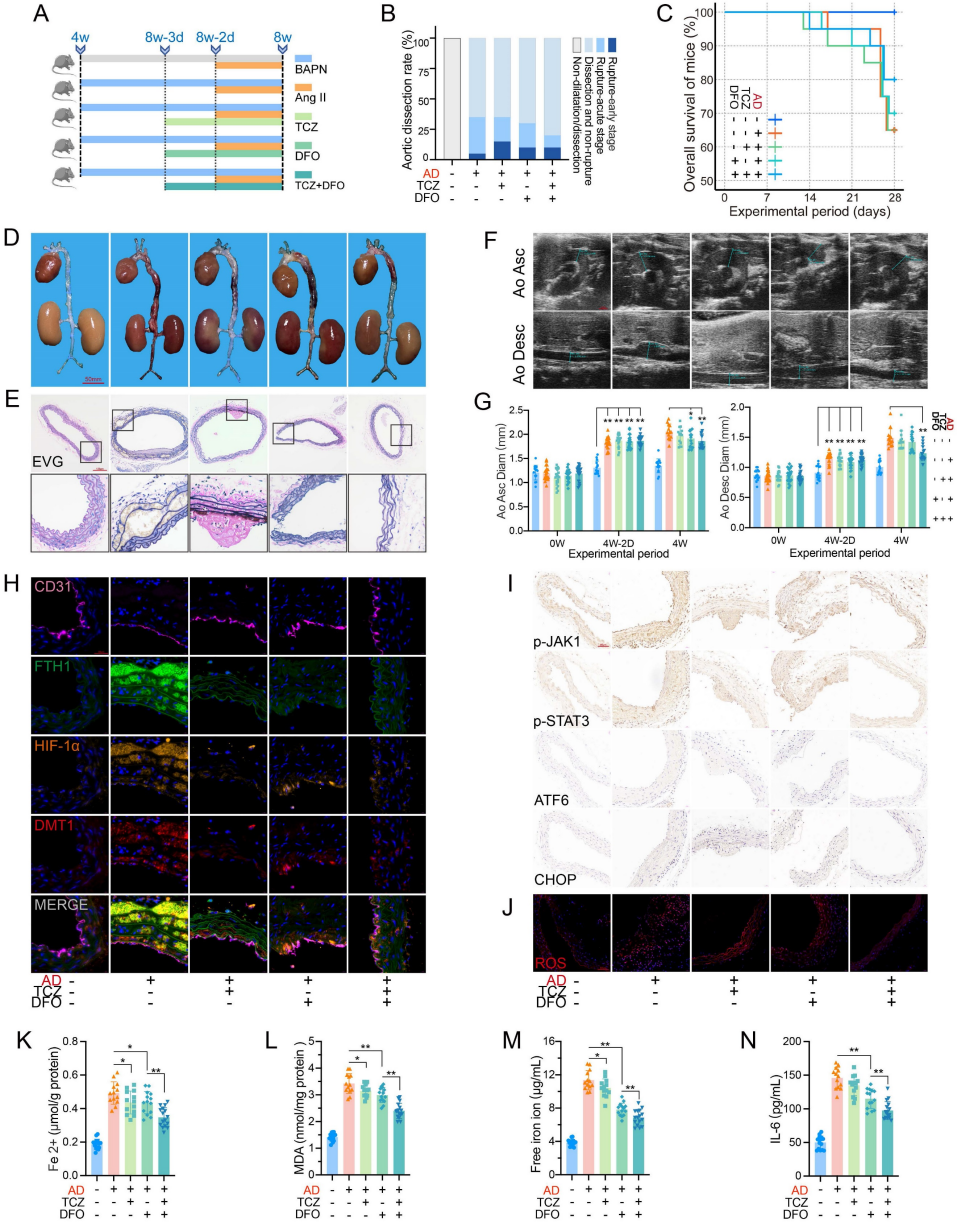
Dissection progression was alleviated by blocking IL-6R and iron ions. (**A**) TCZ (intravenous injection; 1.0 mg/100 μL/day) inhibited IL-6R, and DFO (intraperitoneal injection; 200 mg/kg/day) chelated free iron ions (15 mice in control group; 20 mice in per dissection group to prevent accidental death). (**B**) Aortic dissection and rupture rate in mice. (**C**) Overall survival of mice during the experiment. (**D**) Gross anatomical views of aortas. (**E**) EVG staining of aortic sections. (**F**) Ultrasound images of ascending and descending aortas. (**G**) Change of ascending aorta (Left) and descending aorta (Right) diameters. (**H**) Multi-IF exhibited the protein levels of key genes. (**I**) IHC staining of aortic sections. (**J**) ROS staining of aortic tissues. (**K**, **L**) Contents of Fe^2+^ and MDA in mouse aortic tissues. (**M**, **N**) Contents of free iron ion and IL-6 in mouse serums. Data are presented as representative images or means ± SDs. *P < 0.05, **P < 0.01, ***P < 0.001, ns, not significant.

Before inducing the acute phase, IL-6R signaling was blocked by TCZ, and free iron was chelated by DFO (**Fig. 6A**). Mouse aortas, except for the control group, showed dissection or evident dilatation.

Mice without TCZ or DFO had the most ruptures (30%, 6/20) in the acute phase, and the rate decreased to 10% (2/20) under combined therapy with TCZ and DFO (**Fig. 6B**-**C**). The dissecting aortas were dilated and congested, and EVG staining indicated fiber plate loss and fracture. Combination of TCZ and DFO alleviated the pathological progression (**Fig. 6D**-**E**). The combination therapy obviously inhibited aortic dilatation (**Fig. 6F**-**G**). Multi-IF revealed high FTH1, HIF-1α, and DMT1 expression in dissecting aortas. TCZ markedly downregulated HIF-1α and DMT1 expression. DFO, especially combination therapy, reduced FTH1 expression and iron load (**Fig. 6H**). Similarly, JAK1-STAT3 phosphorylation was mainly downregulated by IL-6R inhibition. DFO and TCZ reduced ERS marker and ROS levels separately, and combination had the greatest effect (**Fig. 6I**-**J**).

TCZ and DFO inhibited Fe^2+^ and MDA in dissecting aortas, with greater repression when combined (**Fig. 6K**-**L**). DFO more potently suppressed serum free iron ions and IL-6 than did TCZ (**Fig. 6M**-**N**). Serum hepcidin, creatinine, Kim-1, and ROS levels were decreased by monotherapy, with better effect in combination (**Fig. S3E-H**). TCZ enhanced the alleviation of DFO on circulating iron overload, and combined therapy reduced the progression and organ damage of the acute dissection.

On gross view, no obvious difference was found in renal sections (**Fig. S3I**). PAS staining indicated glomerular collapse and mesangial hyperplasia in AD mice, which were relieved by TCZ and DFO (**Fig. S3J**). IHC and ROS staining of renal sections were similar to previous aortas, and we speculated that excessive IL-6 and free iron in the acute dissection have similar damage mechanisms to the kidney (**Fig. S3K-L**).

### Iron overload and ERS increase in AD patients

**Figure 7:**
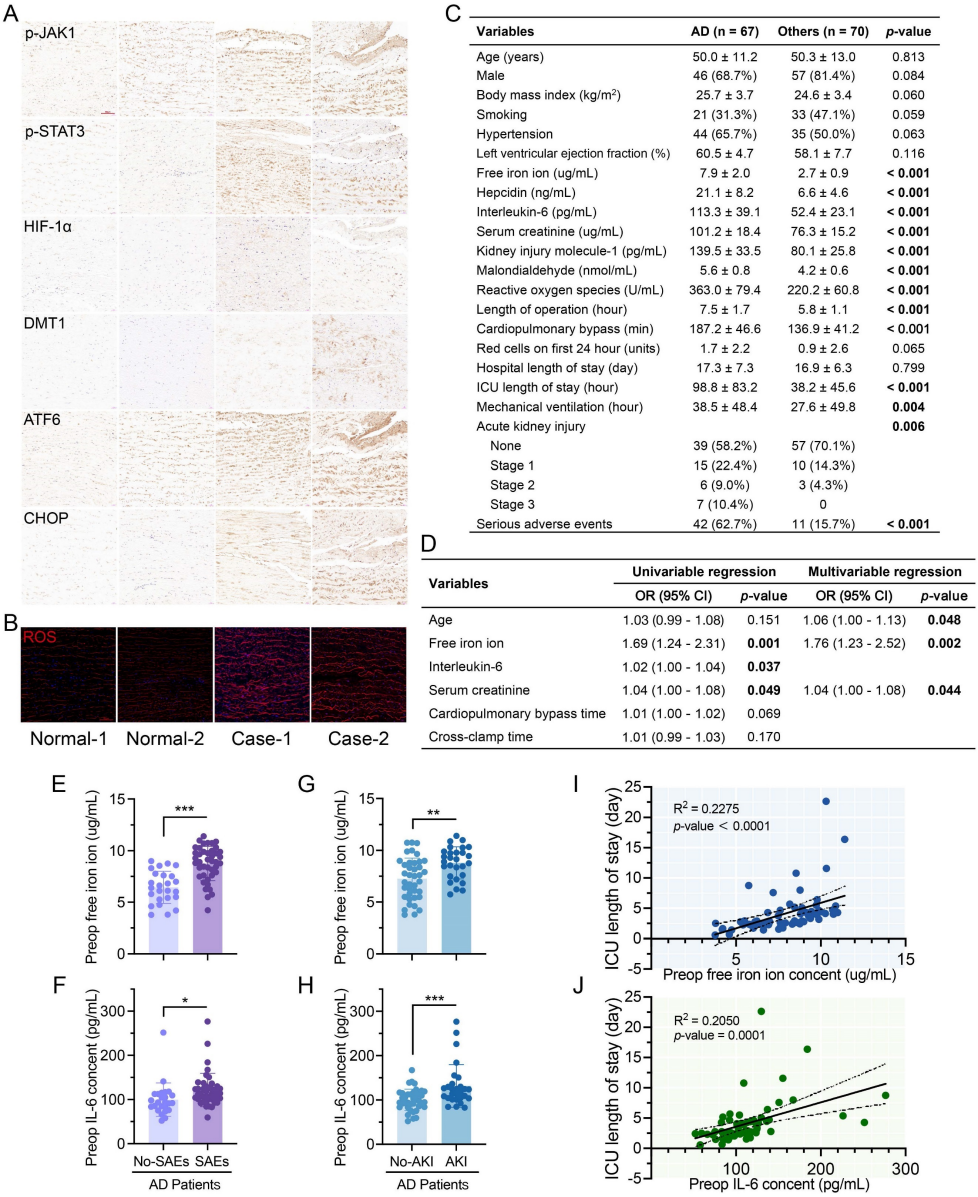
Iron overload and excess IL-6 were dissection characteristics and worsened prognosis. (**A**) IHC staining of patient and transplant donor aortas. (**B**) ROS staining of aortic tissues. (**C**) Clinical data of AD patients (n = 67) and other patients (n = 70) requiring aortic surgery. Variables are displayed as n (%) and mean ± SDs. (**D**) Univariable and multivariable logistic regression of SAEs in AD patients. OR, odds ratio; CI, confidence interval. (**E**, **F**) Preoperative free iron and IL-6 in AD patients with or without SAEs. (**G**, **H**) Preoperative free iron and IL-6 in AD patients with or without AKI. (**I**) Correlation between preoperative free iron ion and ICU stay in AD patients. (**J**) Relationship between the IL-6 and ICU stay in AD patients. Data are presented as representative images or means ± SDs. *P < 0.05, **P < 0.01, ***P < 0.001, ns, not significant.

IHC staining revealed elevated p-JAK, p-STAT3, HIF-1α, DMT1, ATF6, and CHOP levels in AD patient and transplant donor tissues (**Fig. 7A**). ROS levels in ECs between fibroelastic plates were much greater in AD (**Fig. 7B**).

AD patients had similar baseline data (age, sex, body mass index, left ventricular ejection fraction, smoking, and hypertension history) to other patients (**Fig. 7C**, **Table S4**). AD patients had greater preoperative serum iron ion, hepcidin, IL-6, organ damage marker, and hyperoxide levels. Although there were no differences in transfusion volume or hospital stay, AD patients had longer intensive care and mechanical ventilation and more complications.

Levels of free iron at pre-operation (Preop), end of CPB (End-CPB), and postoperation day one (POD#1) were higher in AD (**Fig. S6A**). Hepcidin did not differ from those in others at End-CPB, nor did IL-6 (**Fig. S3B-C**). AD patients had higher ROS and MDA levels, but MDA recovered faster (**Fig. S3D-E**). No decreasing trend in creatinine or Kim-1 was found in AD after surgery (**Fig. S3F-G**).

Increased age, free iron, and creatinine can significantly worsen the postoperative prognosis of AD (**Fig. 7D**, **Table S5**). IL-6 was also significant in univariable logistic analysis. Preoperative free iron and IL-6 were greater in AD patients who developed AKI and SAEs (**Fig. 7E-H**). ICU stay was positively correlated with the preoperative free iron (R^2^ = 0.2275,* P* < 0.001) and IL-6 (R^2^ = 0.2050,* P =* 0.001) (**Fig. 7O-P**). Hospital stay was positively correlated with the free iron ions (R^2^ = 0.2174,* P* = 0.001) but not IL-6 (**Fig. S6H**-**I**).

### IL-6 pathway upregulated DMT1 to sensitize free iron ion-induced ERS

**Figure 8:**
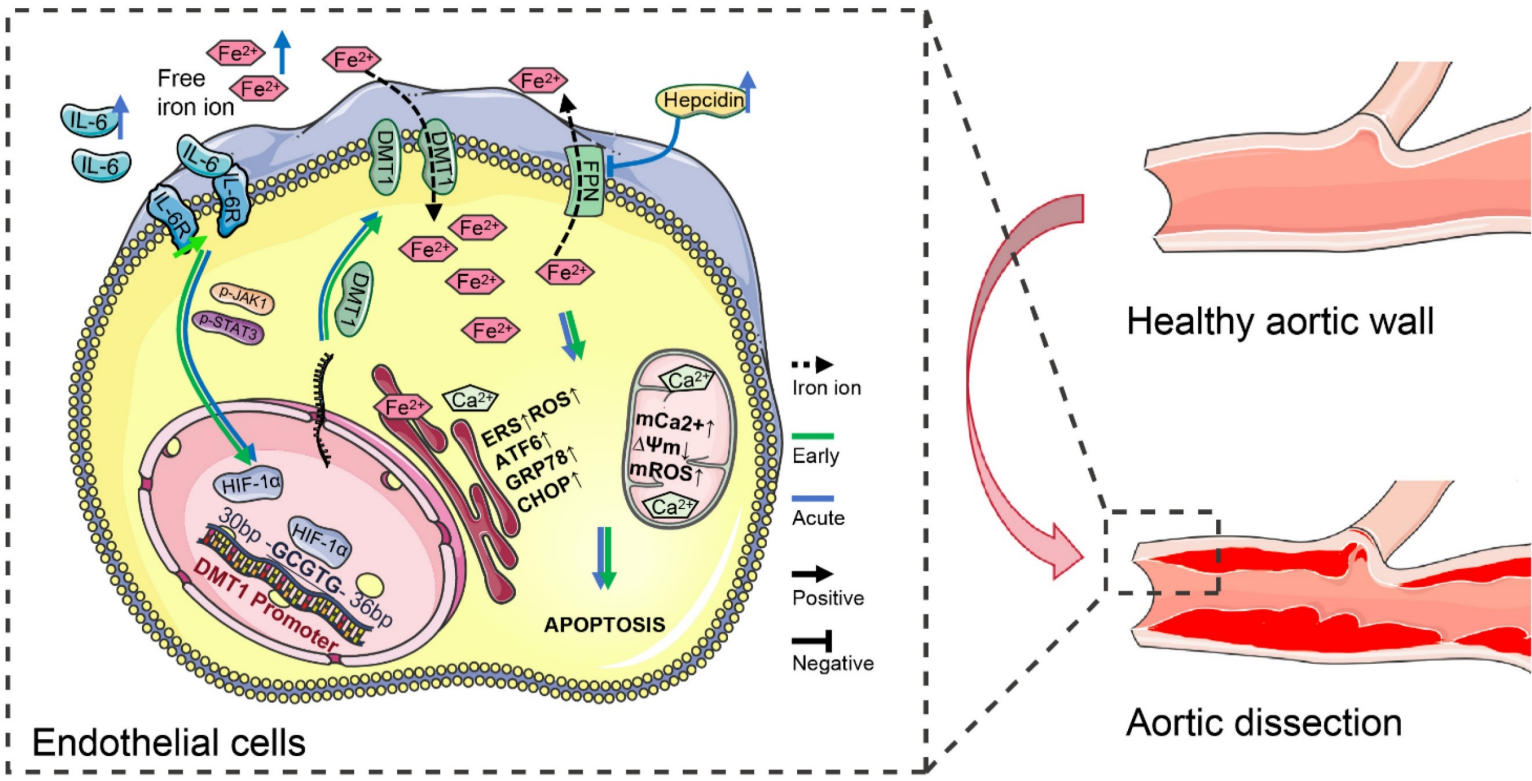
IL-6 pathway upregulated DMT1 to sensitize free iron ion-induced ERS. IL-6 signaling increases HIF-1α first through the JAK1-STAT3 pathway in dissection development, and then HIF-1α transcriptionally upregulates DMT1 expression. High DMT1 expression leads to intracellular iron overload, which induces ERS in ECs. The arrow (→) indicates positive regulation, and the symbol (─┤) indicates negative regulation. Green lines represent early changes, and blue represents the acute phase.

IL-6R overexpression increases HIF-1α first through the JAK1-STAT3 pathway in dissection development. Then HIF-1α binds explicitly to the -GCGTG- sequence in the DMT1 promoter (+30 - +35 bp), transcriptionally upregulating DMT1 expression (**Fig. 8**). High DMT1 expression leads to intracellular iron overload, which induces ERS in HAECs. Excessive IL-6 and free iron ions in the acute phase aggravate this phenomenon, promoting dissection progression and organ damage. Moreover, hepcidin, which increases with circulating IL-6 and free iron ions, inhibits FPN from transferring out iron ions, further exacerbating iron overload.

## Discussion

AD, which can be cured only surgically, is a serious issue in clinical treatment. Therefore, the identification of drug targets for the early and acute phases of AD is critical^1^,^2^. The relationship between iron and cardiovascular disease and perioperative organ damage has been reported^8^,^23^. However, there is still limited research on precisely regulating iron ions in these cells and tissues^8^. This study, for the first time, identified and defined characteristic ECs of AD by scRNA-seq data analysis, which exhibited significant iron overload. The noncanonical iron regulatory axis of the IL-6/JAK1/STAT3 pathway upregulated DMT1 expression by increasing HIF-1α was demonstrated *in vivo* and *in vitro*. High DMT1 then leads to iron accumulation in HAECs, which is the direct cause of inducing ERS and dissection. Beyond that, by comparing clinical data of AD and other aortic disease patients, it was found that high free iron ions, systemic inflammation, and oxidative stress levels were major features of AD patients and correlated with prognosis. The discovery of nonclassical iron regulatory mechanisms and clinical characteristics of AD has guiding value for potential target research and drug treatment strategies.

Iron disorder has been identified as the trigger in the progression of various cardiovascular diseases, whether iron deficiency or iron overload^24^. Understanding iron's metabolism characteristics and transport mechanism in different diseases contributes to the treatment. This study identified the top-expressing FTL in characteristic AECs of AD. Furthermore, FTL and FTH1, essential markers of iron content, increased with EC differentiation 6. Therefore, iron overload is a typical feature of AECs with AD and is involved in the occurrence and progression of dissection. Through intersection and confirmation, the iron homeostasis genes HIF-1α and DMT1 were identified for high expression. As a transporter of iron import, DMT1 is the key to explaining iron overload in AD. DMT1 has been reported with ischemic injury, atherosclerosis, vascular calcification, and dementia, and its association with vascular dissection is a discovery^25^,^26^.

Inflammatory reactions prevent infection from spreading and promote repair, but inflammatory signaling disorders cause metabolic change and dysfunction^27^. Studies have shown that interleukin, tumor necrosis factor (TNF), IFN, and chemokines regulate iron metabolism through the inflammasome, JAK-STAT, and MAPK pathways^28^-^30^. The classical mechanism of IL-6/JAK/STAT signaling promotes the hepcidin release, which acts on FPN to reduce Fe^2+^ efflux^31^,^32^. Perhaps due to negative feedback, AD patients and mice with high circulating free iron ion and hepcidin levels showed low hepcidin expression in aortas by bioinformatics analysis. Obviously, these results do not apply to the self-regulation of iron in ECs with AD.

Our study identified that the JAK/STAT pathway with characteristic enrichment in AD is activated by IL-6R signaling. Furthermore, the IL-6 level was correlated with the content and metabolism of aortic iron, suggesting a potential correlation between the IL-6 pathway and DMT1. The direct relationship between the canonical iron regulatory axis IL-6/JAK/STAT and DMT1 has rarely been reported^33^,^34^. In addition, STAT6 transcriptionally inhibits DMT1 expression to reduce ferroptosis and atherosclerosis^35^. However, the increased HIF-1α expression and signaling may be a bridge for IL-6 signaling to regulate DMT1^36^. This hypothesis was fully verified. Aortic HIF-1α and DMT1 expression and lesions were significantly alleviated in mice after blocking IL-6R, demonstrating the feasibility of drug therapy for relieving AD progression and organ injury^37^-^39^.

The regulation of cytokines on HIF-1α expression and activity is diverse^22^,^40^. TNF-α and IL-1 can even induce and maintain HIF-1α protein under normoxia^41^,^42^. However, in smooth muscle cells, although TNF-α can upregulate HIF-1α mRNA and protein, its activity is low^43^. IFN requires cooperation with cytokines to regulate HIF-1α through a STAT-dependent mechanism^44^. Tissue-specific induction and activation of HIF-1α results in these differences. Our study hypothesized that the JAK/STAT3 directly increases HIF-1α expression. On the other hand, cytokine-induced hypoxia and ERS in microenvironment increase HIF-1α stability^45^. Because the regulation of HIF-1a by IL-6/JAK1/STAT3 has been reported, we did not investigate the mechanism but validated it *in vivo* and *in vitro*^46^-^48^.

DMT1 is involved in the occurrence of many diseases, such as Parkinson's disease^49^,^50^. Cytokines, including TNF-α and IFN-γ, can increase DMT1 expression; conversely, DMT1 dysregulation induces inflammation and ROS^33^,^34^. In general, DMT1 is managed by the iron response protein (IRP)/iron response element (IRE) in an iron-dependent system^3^,^51^. Excessive iron or ROS inhibits the IRP/IRE system and reduces DMT1 expression^51^,^52^. Interestingly, abnormal DMT1 expression suggested other regulations in AD and iron overload progression. Our evidence supported that HIF-1α has direct transcriptional activity on DMT1 in HAECs, which then leads to iron imbalance. Abnormal IL-6R first activates this noncanonical regulatory axis, resulting in iron overload and AD. Excessive systemic IL-6, free iron ion, and hepcidin in the acute phase aggravate this phenomenon, exacerbating progression and organ damage^8^,^32^.

Our results showed that high systemic IL-6 and free iron levels are characteristic of the acute phase of AD patients. In particular, free iron was correlated with patient prognosis. Although changes in free iron during the cardiac surgery perioperative period and their relationship with AKI have long been reported, the underlying causes remain unclear^8^,^23^. In AD mice, serum creatinine, Kim-1, and hyperoxide levels showed similar trends to IL-6 and free iron, and DFO and TCZ treatments were effective. It suggested that IL-6 and iron may damage the kidney through the same non-classical pathway. Finding these regulatory mechanisms is essential for organ protection in AD patients and those requiring cardiac surgery.

During AD development and progression, the noncanonical iron regulatory axis of the IL-6/JAK1/STAT3 pathway upregulated DMT1 expression by increasing HIF-1α first. High DMT1 is the direct cause of iron ions accumulation and ERS in HAECs, which in turn induces AD. Inhibiting IL-6 signaling and chelating acutely elevated free iron ions may be reliable targets for drug research to reduce AD progression and organ injury.

## Supplementary Material

Supplementary figures and tables.

## Abbreviations

AD: aortic dissection
AEC: aortic endothelial cell
AAD: acute aortic dissection
ChIP: chromatin immunoprecipitation
CPB: cardiopulmonary bypass
DEG: differential expression gene
DMT1: divalent metal transporter 1
ERS: endoplasmic reticulum stress
FAC: ferric ammonium citrate
HIF-1α: hypoxia-inducible factor-1α
HAEC: human aortic endothelial cell
IFN: interferon
IL-6R: interleukin-6 receptor
IRP/IRE: iron response protein/Iron response element
Multi-IF: multiple immunofluorescence staining
POD#1: postoperation day one
ROS: reactive oxygen species
TNF: tumor necrosis factor
ZIP-14/8: Zrt/Irt-related proteins 14/8
