# Supplementary material for: IL-6 signaling accelerates iron overload by upregulating DMT1 in endothelial cells to promote aortic dissection
Source: Int J Biol Sci. 2024 Aug 6;20(11):4222–37. doi: 10.7150/ijbs.99511 (PMC11379073; doi:10.7150/ijbs.99511)
Supplement: Supplementary file 1 — Supplementary figures and tables. [file ijbsv20p4222s1.pdf]

# IL-6 signaling accelerates iron overload by upregulating DMT1 in endothelial cells to promote aortic dissection

**Authors:** Qiang Xie[1,2], Jianji Wang[1], Runqiao Li[1], Hao Liu[1], Yongliang Zhong[1], Qinfeng Xu[1], Yipeng Ge[1], Chengnan Li[1], Lizhong Sun[1] and Junming Zhu[1,*]

[1] Department of Cardiovascular Surgery, Beijing Aortic Disease Center, Beijing Anzhen Hospital, Capital Medical University, Beijing 100029, China

[2] Department of Thoracic Surgery, Ruijin Hospital, Shanghai Jiao Tong University School of Medicine, Shanghai 200025, China.

* **Correspondence:** Professor Junming Zhu, MD. Department of Cardiovascular Surgery, Beijing Aortic Disease Center, Beijing Anzhen Hospital, Capital Medical University, 2 Anzhen Avenue, Beijing 100029, China.

**Tel:** +86 13801132276

**E-mail:** anzhenzjm@ccmu.edu.cn; anzhenzjm@163.com

**Supplementary Figure count:** 6

**Supplementary Table count:** 5

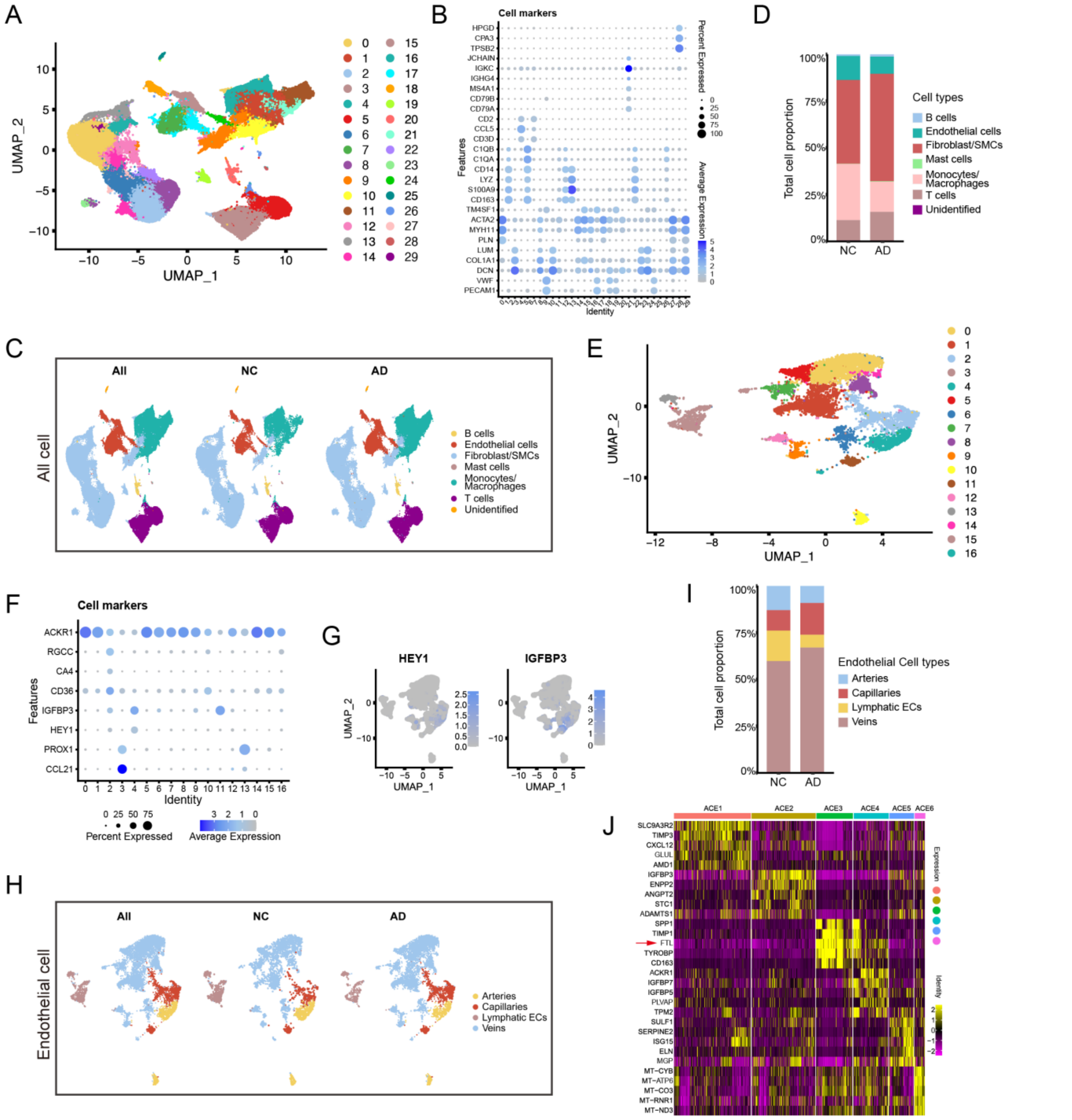


**Fig. S1.** Dimensionality reduction and clustering of aortic cells in scRNA-seq data. (**A**) UMAP plot of all cells and separate clusters. (**B**) Gene expression profiles of selected markers in separate clusters. (**C**) UMAP plot of seven cell types in the aortas. (**D**) Proportion of different cell types in the aortas. (**E**) Separate clusters of all endothelial cells (ECs). (**F**) Gene expression profiles of selected markers in separate clusters of ECs. (**G**) UMAP plot of the expression of aortic endothelial cell (AEC) marker genes (*HEY1* and *IGFBP3*). (**H**) UMAP of EC types in the aortas. (**I**) Proportion of different ECs. (**J**) Heatmap showing the top five genes in AECs.

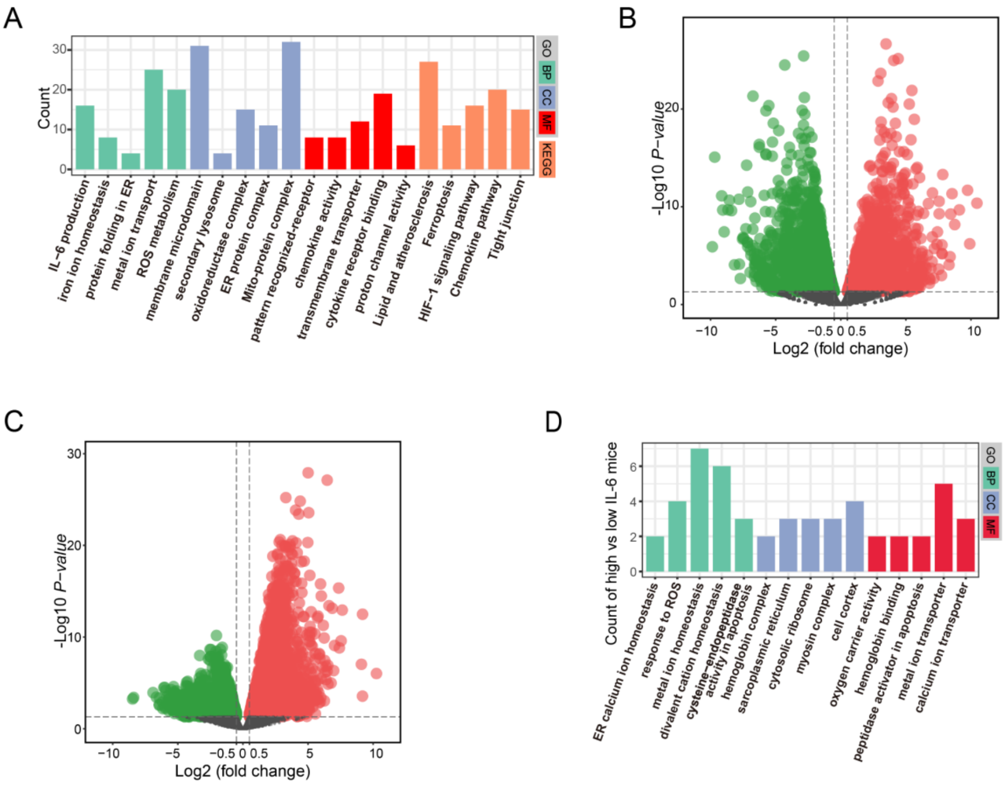


**Fig. S2.** Volcano plot and enrichment analysis of differentially expressed genes (DEGs) in scRNA-seq and bulk RNA-seq. (**A**) GO and KEGG analyses of DEGs in aortic endothelial cell 3 of scRNA-seq. (**B**) Volcano plot of bulk RNA-seq showing DEGs in aortas of dissection patients and transplant donors. (**C**) Volcano plot showing DEGs in the aortas of six control and six acute AD mice. (**D**) GO and KEGG analyses of DEGs between acute control and AD mouse aortas.

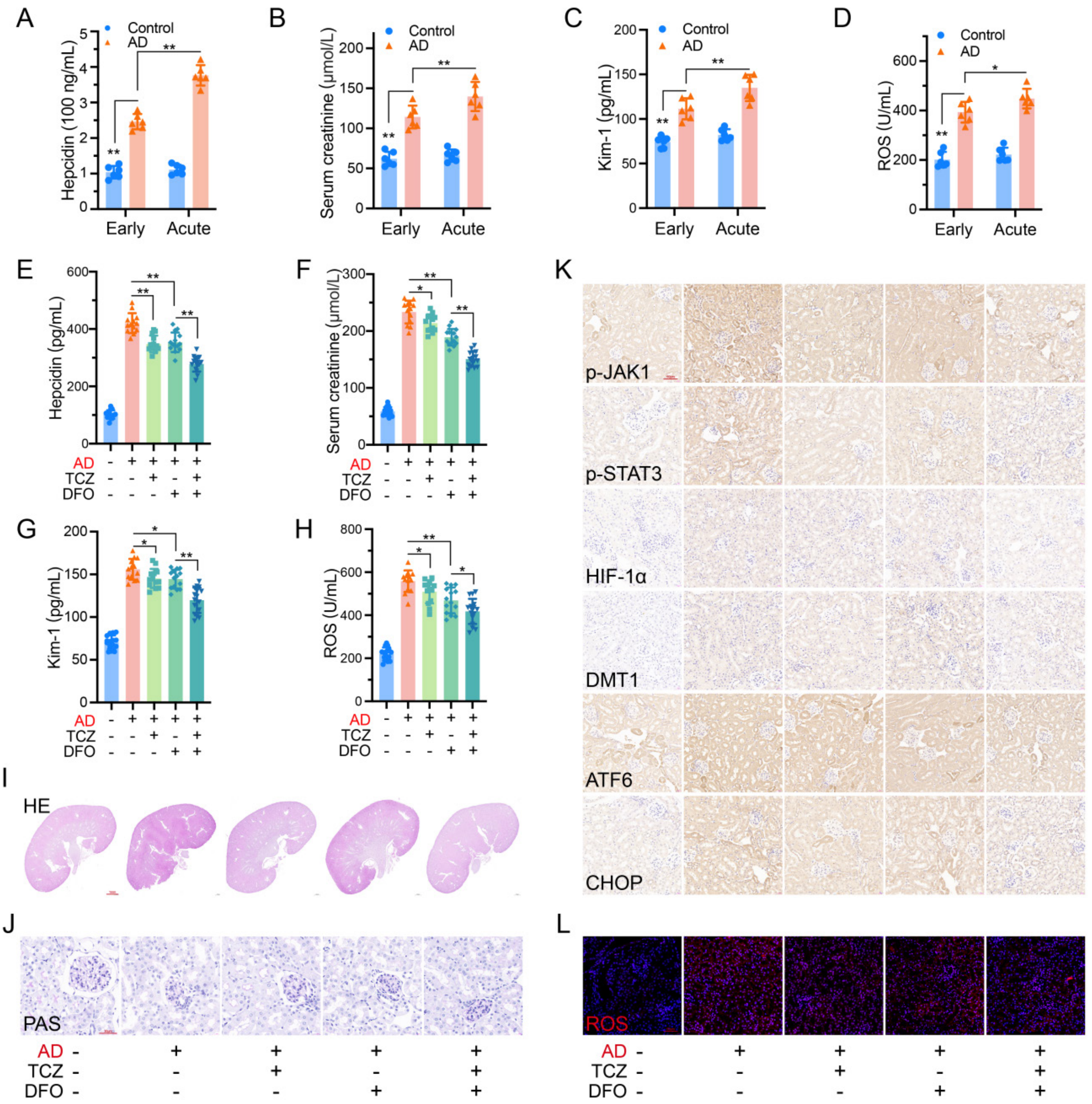


**Fig. S3.** Serum biomarker levels and renal section staining in mice with aortic dissection. Contents of serum hepcidin (**A**), creatinine (**B**), Kim-1 (**C**), and ROS (**D**) in the first animal experiment. TCZ and DFO inhibited serum levels of hepcidin (**E**), creatinine (**F**), Kim-1 (**G**), and ROS (**H**). (**I**) HE staining of coronal sections of mouse kidneys. (**J**) PAS staining of renal sections. (**K**) IHC staining of renal sections. (**L**) ROS staining of renal tissues.

Data are presented as representative images or means ± SDs. *$P < 0.05$, **$P < 0.01$, ns, not significant.

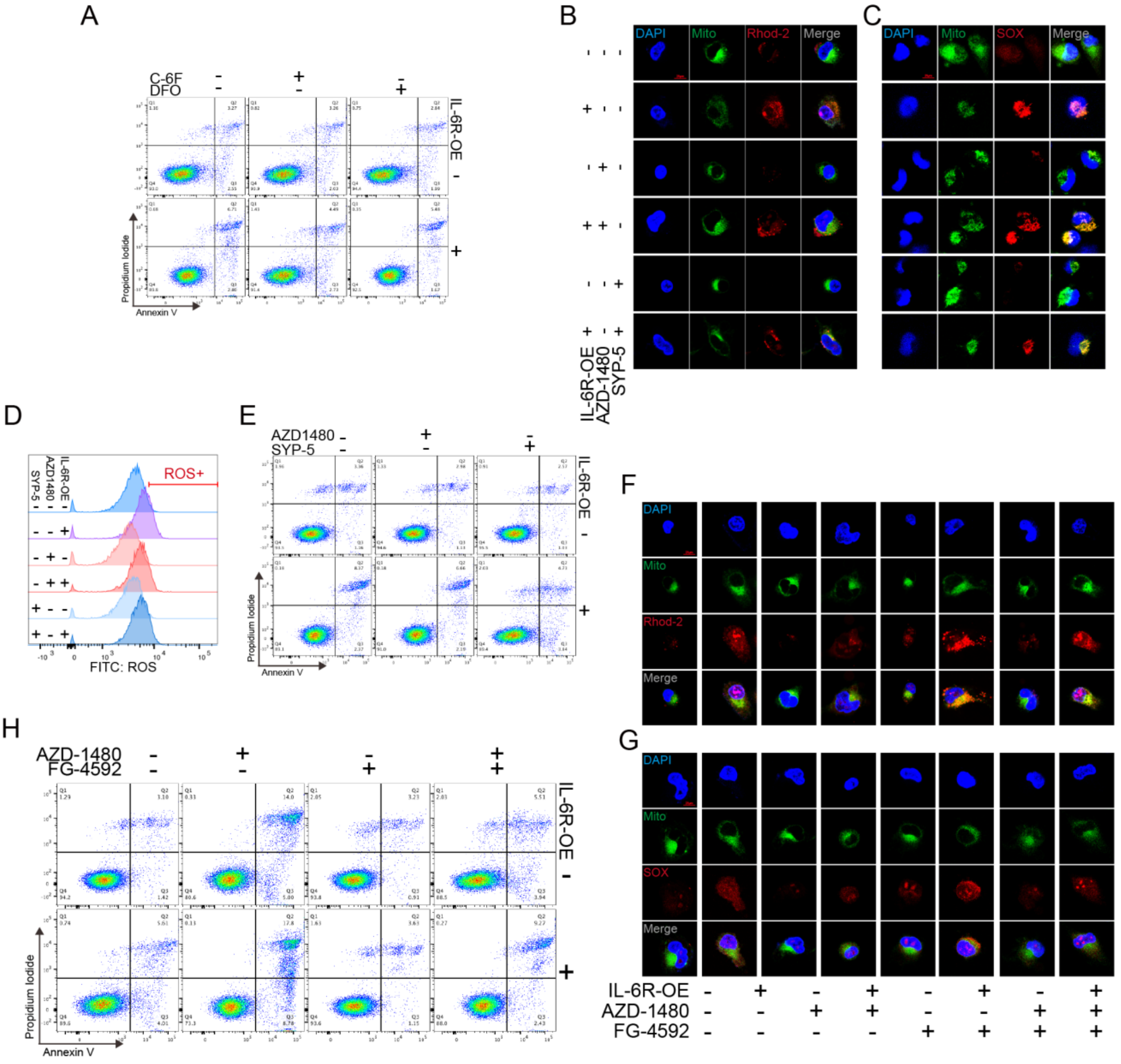


**Fig. S4.** IL-6R overexpression induces ERS and apoptosis in HAECs. (**A**) C-6F and DFO reduced apoptosis in HAECs. (**B**) Confocal images of mito-$Ca^{2+}$ fluorescence staining. (**C**) Confocal images of mitoSOX fluorescence staining. (**D**) AZD1480 and SYP-5 reduced ROS in HAECs. (**E**) AZD1480 and SYP-5 reduced apoptosis in HAECs. (**F**) Fluorescence images of mito-$Ca^{2+}$. (**G**) MitoSOX fluorescence staining. (**H**) Apoptosis of HAECs was detected by flow cytometry. Data are presented as representative images.

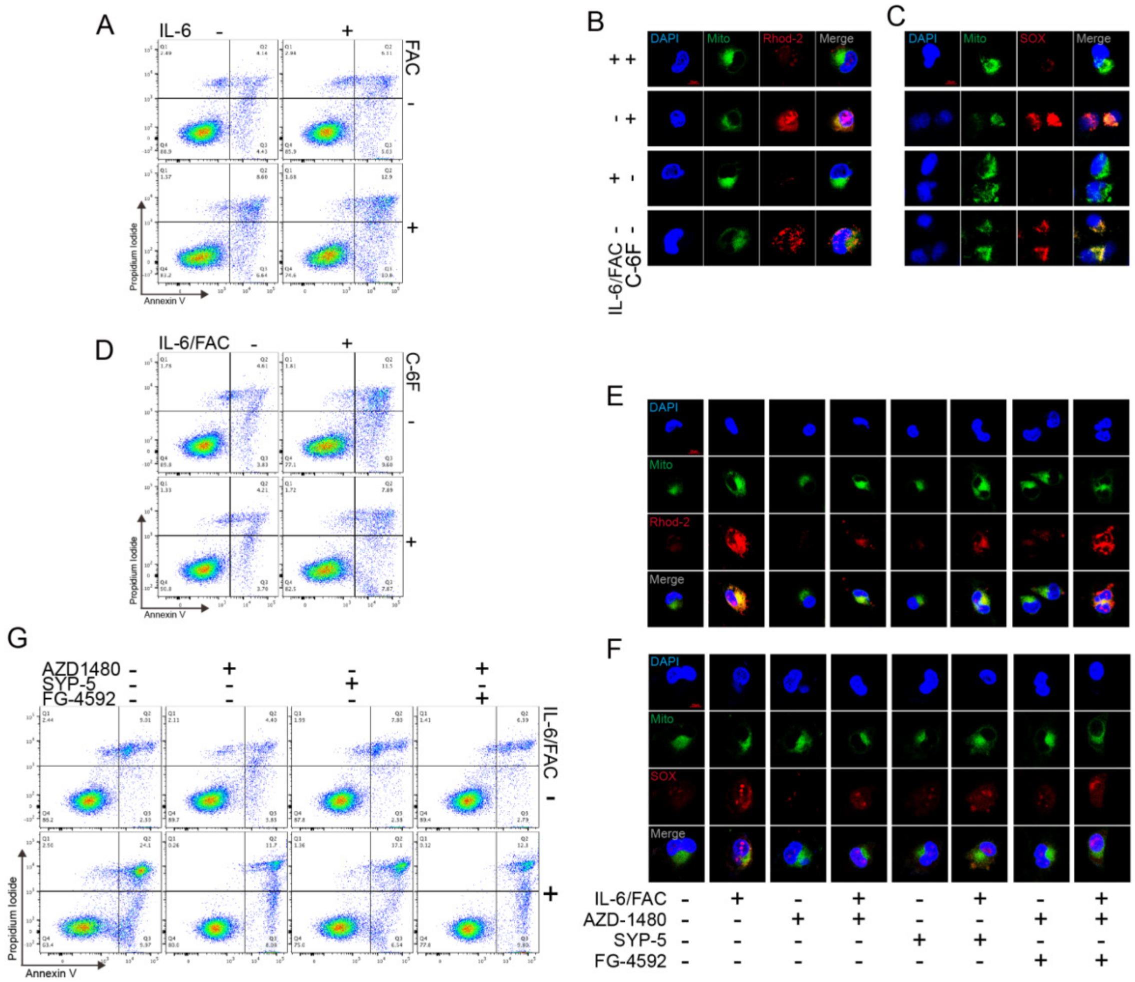


**Fig. S5.** Excessive IL-6 sensitized FAC-induced ERS and apoptosis in HAECs. (**A**) IL-6 sensitized FAC-induced apoptosis in HAECs. (**B**) Confocal images of mito-$Ca^{2+}$ fluorescence staining. (**C**) Confocal images of mitoSOX fluorescence staining. (**D**) C-6F reduced apoptosis induced by IL-6 and FAC in HAECs. (**E**) Fluorescence images of mito-$Ca^{2+}$. (**F**) MitoSOX fluorescence staining. (**G**) Apoptosis of HAECs was detected by flow cytometry. Data are presented as representative images or means ± SDs.

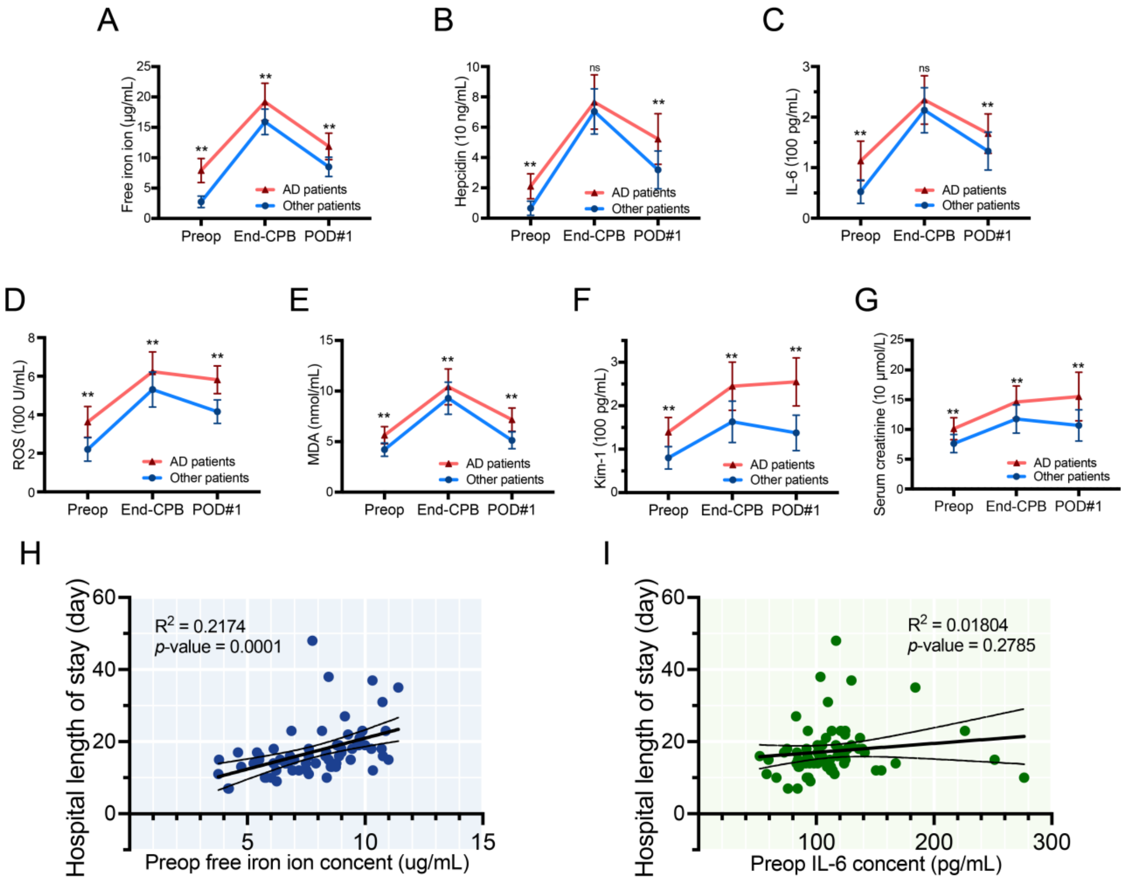


**Fig. S6.** Changes in perioperative serum biomarkers and the correlation between biomarkers and prognosis. Levels of serum iron ion (**A**), hepcidin (**B**), IL-6 (**C**), ROS (**D**), MDA (**E**), Kim-1 (**F**), and creatinine (**G**) in aortic dissection (AD) patients and other patients requiring aortic surgery at pre-operation (Preop), end of cardiopulmonary bypass (End-CPB), and postoperation day one (POD#1). (**H**) Correlation between preoperative free iron ion and hospital stay in AD patients. (**I**) Relationship between preoperative IL-6 and hospital stay in dissection patients. Data are presented as representative images or means ± SDs. *$P < 0.05$, **$P < 0.01$, ns, not significant.

**Table S1.** The primers sequence information.

| Categories | Forward (5'--3') | Reverse (5'--3') |
|---|---|---|
| **RT-PCR assay** | | |
| **Human** | | |
| *FTH1* | -CCAGAACTACCACCAGGACTC- | -GAAGATTCGGCCACCTCGTT- |
| *FTL* | -CAGCCTGGTCAATTTGTACCT- | -GCCAATTCGCGGAAGAAGTG- |
| *HIF-1α* | -GAACGTCGAAAAGAAAAGTCTCG- | -CCTTATCAAGATGCGAACTCACA- |
| *SLC39A8* | -ATGCTACCCAAATAACCAGCTC- | -ACAGGAATCCATATCCCCAAACT- |
| *SOD2* | -GGAAGCCATCAAACGTGACTT- | -CCCGTTCCTTATTGAAACCAAGC- |
| *HMOX1* | -AAGACTGCGTTCCTGCTCAAC- | -AAAGCCCTACAGCAACTGTCG- |
| *DMT1* | -CGGGTGCTGGAGCTATACAG- | -CGACAGTGTTGACATCAATGGC- |
| *PTPRC* | -ACCACAAGTTTACTAACGCAAGT- | -TTTGAGGGGGATTCCAGGTAAT- |
| *IL6R* | -CCCCTCAGCAATGTTGTTTGT- | CTCCGGGACTGCTAACTGG- |
| *LYN* | -TGCAGAGGGAATGGCATACAT- | -TGACTCGGAGACCAGAACATTAG- |
| *CSF1R* | -CTGCCCAGATCGTGTGCTC- | -AGGTTGAGGGTCAGGACTTTTT- |
| *GAPDH* | -CGGGAAGCTTGTCATCAATGG- | -GGCAGAGATGATGACCCTTT- |
| **Mouse** | | |
| *Ptprc* | -GTTTTCGCTACATGACTGCACA- | -AGGTTGTCCAACTGACATCTTTC- |
| *Il6r* | -GCCACCGTTACCCTGATTTG- | -TCCTGTGGTAGTCCATTCTCTG- |

| Categories | Forward (5'--3') | Reverse (5'--3') |
|---|---|---|
| *Lyn* | -ATTGTGGTGGCCTTATACCCT- | -ACCATTCCCCATGCTCTTCTA- |
| *Csf1r* | -TGTCATCGAGCCTAGTGGC- | -GGTCCAAGGTCCAGTAGGG- |
| *Gapdh* | -AGGTCGGTGTGAACGGATTTG- | -GGGGTCGTTGATGGCAACA- |
| **ChIP assay** | | |
| DMT1-Site1 | -AGGGACCCAGATGAAAGGTCTCTTT- | -GGTTCACGCCTGTAATCCCAGCA- |
| DMT1-Site2 | -TGTTTAAGGTGTACAAGATGGAGG- | -AATCCTTGTAGTAGGCTGGCAC- |
| DMT1-Site3 | -TCCCCTCCTCCCAGATTGAG- | -AGACATCAGAGCCACGCAAA- |
| **Pulldown assay (biotin-labeling)** | | |
| DMT1-Site1-probe | -AGGGACCCAGATGAAAGGTCT- | -ACACCTTAAACAATTCCCTTCCATT- |
| DMT1-Site2-probe | -TGTTTAAGGTGTACAAGATGGAGG- | -AATCCTTGTAGTAGGCTGGCAC- |
| DMT1-Site3-probe | -AAACTTCTAGAAAACTGCCTGC- | -TAACCCCAAGCCTGACAGTG- |
| **Luciferase plasmids construction** | | |
| DMT1-WT -pGL3-Basic | -AGGTACCGAGCTCTTACGCGTAATTCTCCTGCCTGAGCCTCCCA- | -GCTTACTTAGATCGCAGATCTGGTGCAGAACTAGTTTGACTTTCCCAATGC- |
| DMT1-Mut-1 | -ACCACTGTGCCTGGCCGCAA- | -TTGCGGCCAGGCACAGTGGTTCTGTAATCCCAGCACTTTG- |
| DMT1-Mut-2 | -GCTGGGATTGCAGAATTTTTGTGCCAGCCTACTAC- | -AAAAATTCTGCAATCCCAGCACTTTGG- |
| DMT1-Mut-3 | -CCAAGTCCGGAGTTTGCTCTGATGTCTGCTCGTGG- | -AGAGCAAACTCCGGACTTGGTGC- |

**Abbreviations:** RT-PCR, reverse transcription-polymerase chain reaction assay; ChIP, chromatin immunoprecipitation; WT, wild type plasmid; Mut, mutant plasmid.

**Table S2.** The antibodies used information.

| Categories | Usage and dosage | Product codes | Sources |
|---|---|---|---|
| **Primary antibodies** | | | |
| FTH | WB 1:200<br>IHC 1:100<br>IF 1:50 | sc-376594 | Santa Cruz, California, USA |
| FTL | WB 1:200<br>IHC 1:100<br>IF 1:50 | sc-74513 | Santa Cruz, California, USA |
| IL-6R | WB 1:200<br>IHC 1:100<br>IF 1:50 | sc-373708 | Santa Cruz, California, USA |
| HIF-1α | WB 1:1000<br>IHC 1:500<br>IF 1:100<br>ChIP 2 ug | #36169 | CST, Massachusetts, USA |
| DMT1 | WB 1:1000<br>IHC 1:200<br>IF 1:100 | 20507-1-AP | Proteintech, Illinois, USA |
| CD31 | IF 1:100 | 11265-1-AP | Proteintech, Illinois, USA |
| p-JAK1 | WB 1:1000<br>IHC 1:200 | SAB4300123 | SIGMA, Missouri, USA |
| p-STAT3 | WB 1:1000<br>IHC 1:200 | #9145 | CST, Massachusetts, USA |
| ATF6 | WB 1:1000<br>IHC 1:200 | 24169-1-AP | Proteintech, Illinois, USA |
| GRP78 | WB 1:1000<br>IHC 1:200 | 11587-1-AP | Proteintech, Illinois, USA |
| CHOP | WB 1:1000<br>IHC 1:200 | 15204-1-AP | Proteintech, Illinois, USA |
| Caspase 12 | WB 1:500 | #58208 | CST, Massachusetts, USA |
| Cleaved-caspase 3 | WB 1:500 | #9661 | CST, Massachusetts, USA |
| GAPDH | WB 1:10000 | 10494-1-AP | Proteintech, Illinois, USA |
| Histone H3 | WB 1:2000 | 17168-1-AP | Proteintech, Illinois, USA |
| Control Rabbit IgG | ChIP 2 ug | #2729 | CST, Massachusetts, USA |

| Categories | Usage and dosage | Product codes | Sources |
|---|---|---|---|
| **Secondary antibodies** | | | |
| IRDye® 800CW Goat anti-Mouse IgG (H+L) | WB 1:20000 | 926-32210 | LI-COR, Nebraska, USA |
| IRDye® 800CW Goat anti-Rabbit IgG (H+L) | WB 1:20000 | 926-32211 | LI-COR, Nebraska, USA |
| Donkey anti-Mouse IgG (H+L) Alexa Fluor™ 488 | IF 1:200 | AB_2556542 | Thermo Fisher Scientific, Massachusetts, USA |
| Goat anti-Mouse IgG (H+L) Alexa Fluor™ 594 | IF 1:200 | AB_2556547 | Thermo Fisher Scientific, Massachusetts, USA |
| Donkey anti-RabbitIgG (H+L) Alexa Fluor™ 594 | IF 1:200 | AB_2556549 | Thermo Fisher Scientific, Massachusetts, USA |
| Goat anti-Rabbit IgG (H+L) Alexa Fluor™ 488 | IF 1:200 | AB_2556544 | Thermo Fisher Scientific, Massachusetts, USA |

**Abbreviations:** WB, western blot; IHC, immunohistochemistry; IF, immunofluorescence; ChIP, chromatin immunoprecipitation.

**Table S3.** The enzyme-linked immunosorbent assay kits used information.

| Categories | Product codes | Sources |
|---|---|---|
| **Human** | | |
| Free iron ion (Non-transferrin binds iron) | MM-60946H1 | Mmbiology, Jiangsu, China |
| Interleukin-6 | MM-0049H1 | Mmbiology, Jiangsu, China |
| Malonaldehyde | MM-2037H1 | Mmbiology, Jiangsu, China |
| Reactive oxygen species | MM-1893H1 | Mmbiology, Jiangsu, China |
| Kidney injury molecule-1 | MM-0647H1 | Mmbiology, Jiangsu, China |
| Creatinine | MM-13833H1 | Mmbiology, Jiangsu, China |
| **Mouse** | | |
| Free iron ion (Non-transferrin binds iron) | MM-46281M1 | Mmbiology, Jiangsu, China |
| Interleukin-6 | MM-0163M1 | Mmbiology, Jiangsu, China |
| Malonaldehyde | MM-0897M1 | Mmbiology, Jiangsu, China |
| Reactive oxygen species | MM-43700M1 | Mmbiology, Jiangsu, China |
| Kidney injury molecule-1 | MM-0318M1 | Mmbiology, Jiangsu, China |
| Creatinine | MM-0693M1 | Mmbiology, Jiangsu, China |

**Table S4.** Clinical details of aortic dissection and other patients.

| **Variables** | **AD (n = 67)** | **Others (n = 70)** | ***p*-value** |
|---|---|---|---|
| **Preoperative variables** | | | |
| Age (years) | 50.0 ± 11.2 | 50.3 ± 13.0 | 0.813 |
| Male | 46 (68.7%) | 57 (81.4%) | 0.084 |
| Height (cm) | 169.6 ± 7.7 | 170.4 ± 7.6 | 0.230 |
| Weight (kg) | 74.0 ± 12.2 | 71.6 ± 11.6 | 0.244 |
| Body mass index (kg/$m^2$) | 25.7 ± 3.7 | 24.6 ± 3.4 | 0.060 |
| Smoking | 21 (31.3%) | 33 (47.1%) | 0.059 |
| Hypertension | 44 (65.7%) | 35 (50.0%) | 0.063 |
| Left ventricular ejection fraction (%) | 60.5 ± 4.7 | 58.1 ± 7.7 | 0.116 |
| Free iron ion (ug/mL) | 7.9 ± 2.0 | 2.7 ± 0.9 | **< 0.001** |
| Interleukin-6 (pg/mL) | 113.3 ± 39.1 | 52.4 ± 23.1 | **< 0.001** |
| Serum creatinine (ug/mL) | 101.2 ± 18.4 | 76.3 ± 15.2 | **< 0.001** |
| Hepcidin (ng/mL) | 21.1 ± 8.2 | 6.6 ± 4.6 | **< 0.001** |
| Kidney injury molecule-1 (pg/mL) | 139.5 ± 33.5 | 80.1 ± 25.8 | **< 0.001** |
| Malondialdehyde (nmol/mL) | 5.6 ± 0.8 | 4.2 ± 0.6 | **< 0.001** |
| Reactive oxygen species (U/mL) | 363.0 ± 79.4 | 220.2 ± 60.8 | **< 0.001** |
| **Operation-related variables** | | | |
| Length of operation (hour) | 7.5 ± 1.7 | 5.8 ± 1.1 | **< 0.001** |
| Cardiopulmonary bypass time (min) | 187.2 ± 46.6 | 136.9 ± 41.2 | **< 0.001** |
| Cross-clamp time (min) | 105.3 ± 28.5 | 89.8 ± 27.5 | **0.003** |
| Assisted circulation time (min) | 73.9 ± 28.4 | 39.0 ± 21.7 | **< 0.001** |
| **Postoperative variables** | | | |
| Packed red cells on first 24h (units) | 1.9 ± 2.2 | 0.7 ± 1.9 | **< 0.001** |
| Frozen plasma on first 24h (100 mL) | 0.9 ± 1.9 | 0.4 ± 1.3 | 0.067 |
| Platelet concentrate on first 24h (units) | 0.1 ± 0.3 | 0 | **0.006** |
| Packed red cells (units) | 4.3 ± 5.7 | 1.2 ± 3.6 | **< 0.001** |

| Variables | AD (n = 67) | Others (n = 70) | *p*-value |
|---|---|---|---|
| **Postoperative variables** | | | |
| Fresh frozen plasma (100 mL) | 1.6 ± 3.6 | 0.7 ± 2.6 | **0.049** |
| Platelet concentrate (units) | 0.4 ± 0.8 | 0.1 ± 0.7 | **< 0.001** |
| Hospital length of stay (day) | 17.3 ± 7.3 | 16.9 ± 6.3 | 0.799 |
| ICU length of stay (hour) | 98.8 ± 83.2 | 38.2 ± 45.6 | **< 0.001** |
| Prolonged ICU of stay (≥ 3 days) | 32 (47.8%) | 7 (10.0%) | **< 0.001** |
| Mechanical ventilation (hour) | 38.5 ± 48.4 | 27.6 ± 49.8 | **0.004** |
| Mechanical ventilation (≥ 24 hours) | 35 (52.2%) | 8 (11.4%) | **< 0.001** |
| In-hospital mortality | 2 (3.0%) | 1 (1.4%) | 0.534 |
| Renal replacement therapy | 5 (7.5%) | 0 | 0.061 |
| Acute kidney injury | 28 (41.8%) | 13 (18.6%) | **0.003** |
| Acute kidney injury | | | **0.006** |
| None | 39 (58.2%) | 57 (70.1%) | |
| Stage 1 | 15 (22.4%) | 10 (14.3%) | |
| Stage 2 | 6 (9.0%) | 3 (4.3%) | |
| Stage 3 | 7 (10.4%) | 0 | |
| Extracorporeal membrane oxygenation | 0 | 2 (2.9%) | 0.497 |
| Stroke | 7 (10.4%) | 1 (1.4%) | 0.059 |
| Paraplegia | 3 (4.5%) | 0 | 0.228 |
| Re-exploration for bleeding | 2 (5.0%) | 2 (2.9%) | 0.228 |
| Serious adverse events | 42 (62.7%) | 11 (15.7%) | **< 0.001** |

**Footnote:** Variables are displayed as n (%) and mean ± standard deviation. The *p*-values with statistical significance are bolded.

**Abbreviations:** ICU, intensive care unit.

**Table S5.** Univariable logistic regression of serious adverse events.

| **Variables** | **OR (95% CI)** | ***p*-value** |
|---|---|---|
| Age | 1.03 (0.99 - 1.08) | 0.151 |
| Male | 1.29 (0.44 - 3.80) | 0.649 |
| Body mass index | 0.99 (0.86 - 1.13) | 0.869 |
| Smoking | 0.71 (0.25 - 2.04) | 0.527 |
| Hypertension | 1.49 (0.53 - 4.18) | 0.452 |
| Left ventricular ejection fraction | 1.01 (0.91 - 1.13) | 0.830 |
| Free iron ion | 1.69 (1.24 - 2.31) | **0.001** |
| Interleukin-6 | 1.02 (1.00 - 1.04) | **0.037** |
| Serum creatinine | 1.04 (1.00 - 1.08) | **0.049** |
| Hepcidin | 1.04 (0.97 - 1.10) | 0.266 |
| Kidney injury molecule-1 | 1.00 (0.98 - 1.01) | 0.584 |
| Malondialdehyde | 0.90 (0.50 - 1.61) | 0.717 |
| Reactive oxygen species | 1.00 (0.99 - 1.00) | 0.549 |
| Cardiopulmonary bypass time | 1.01 (1.00 - 1.02) | 0.069 |
| Cross-clamp time | 1.01 (0.99 - 1.03) | 0.170 |

**Footnote:** Preoperative and operation-related variables with *p*-values < 0.20 in univariable logistic regression were considered candidates for multivariable regression. The *p*-values with statistical significance are bolded.

**Abbreviations:** OR, odds ratio; CI, confidence interval.
